# Correction: Phenology of *Trichodesmium* spp. blooms in the Great Barrier Reef lagoon, Australia, from the ESA-MERIS 10-year mission

**DOI:** 10.1371/journal.pone.0213559

**Published:** 2019-03-05

**Authors:** The PLOS ONE Staff

## Notice of republication

This article was republished on February 25, 2019 to correct for errors introduced during the typesetting process. The publisher apologizes for the errors. Please download this article again to view the correct version. The originally published, uncorrected article and the republished, corrected article are provided here for reference.

## Supporting information

S1 File Originally published, uncorrected article. (PDF)

S2 File Republished, corrected article. (PDF)
