# Supplementary figures and images for: Phenology of Trichodesmium spp. blooms in the Great Barrier Reef lagoon, Australia, from the ESA-MERIS 10-year mission
Source: PLoS One. 2018 Dec 14;13(12):e0208010. doi: 10.1371/journal.pone.0208010 (PMC6294392; doi:10.1371/journal.pone.0208010)

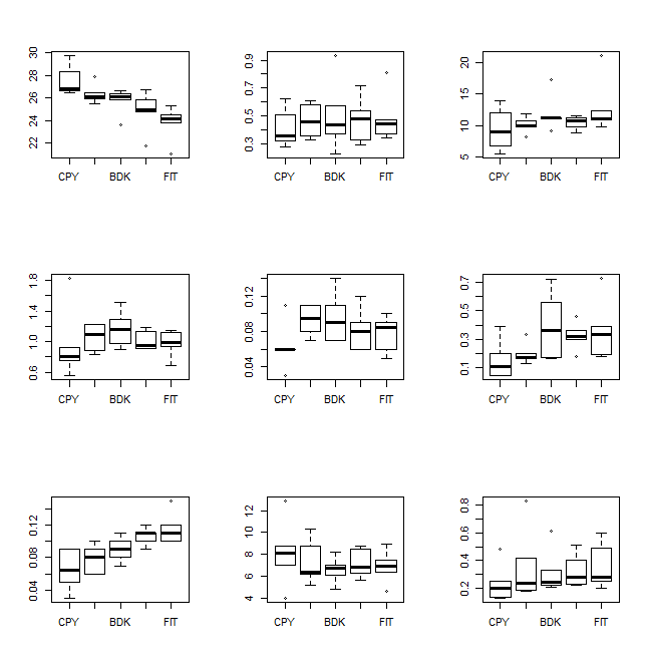

Supplement: S1 Fig — Based on averaged values from the period 2002 to 2013 for temperature, chlorophyll-a (Chl-a), particulate organic carbon (POC), nitrogen (PN), and phosphate (PP), dissolved inorganic nitrogen (DIN) and organic phosphorus (DOP), and total dissolved nitrogen (TDN) and phosphorus (TDP). (TIF) [file pone.0208010.s001.tif]

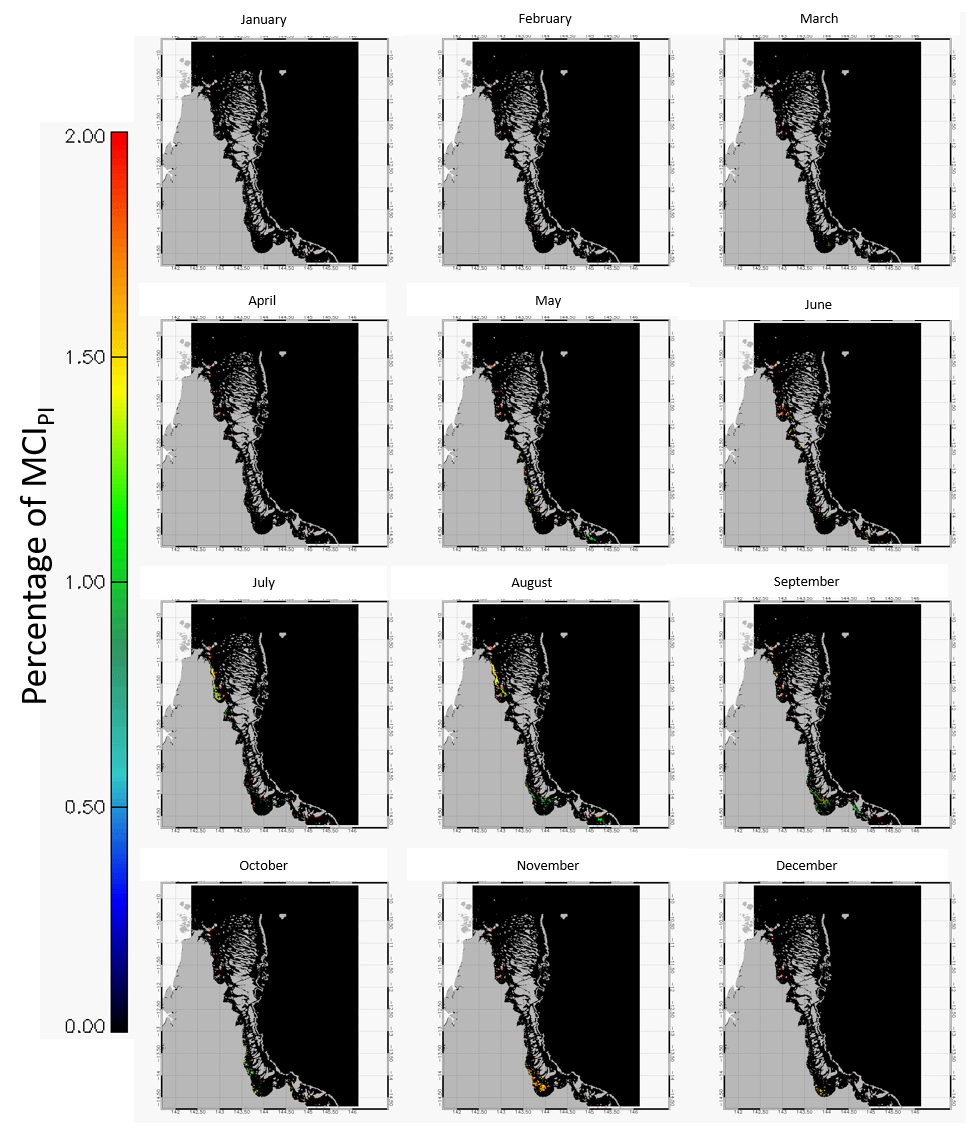

Supplement: S4 Fig — (TIF) [file pone.0208010.s004.tif]

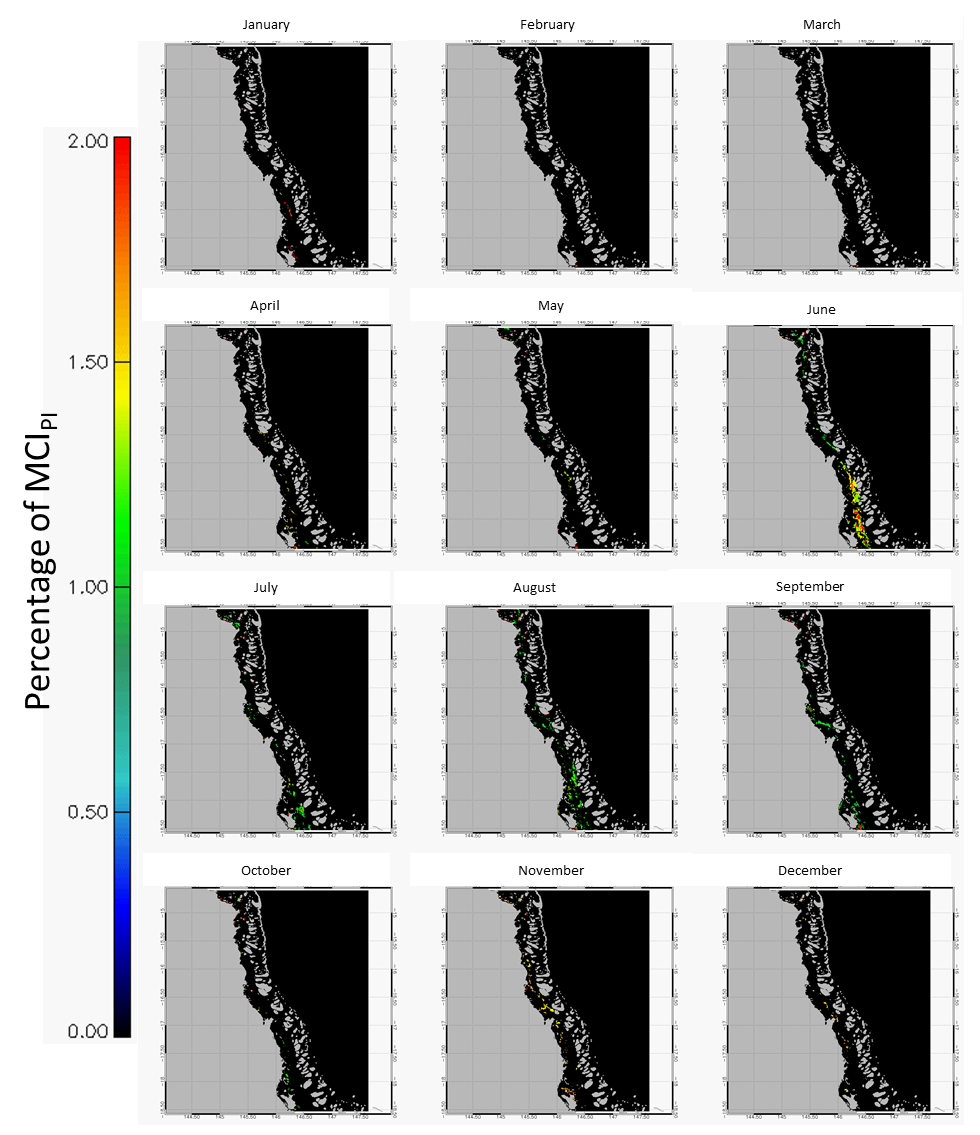

Supplement: S5 Fig — (TIF) [file pone.0208010.s005.tif]

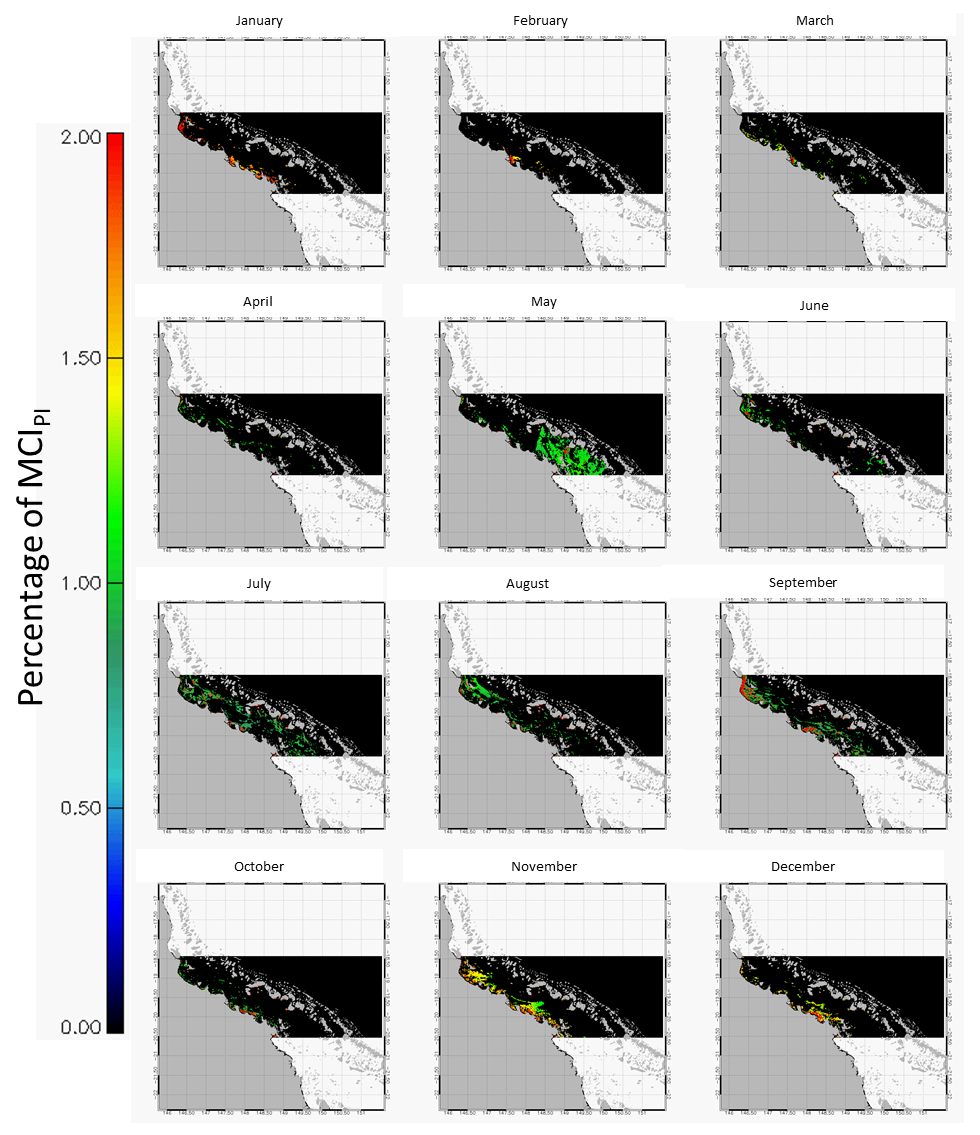

Supplement: S6 Fig — (TIF) [file pone.0208010.s006.tif]

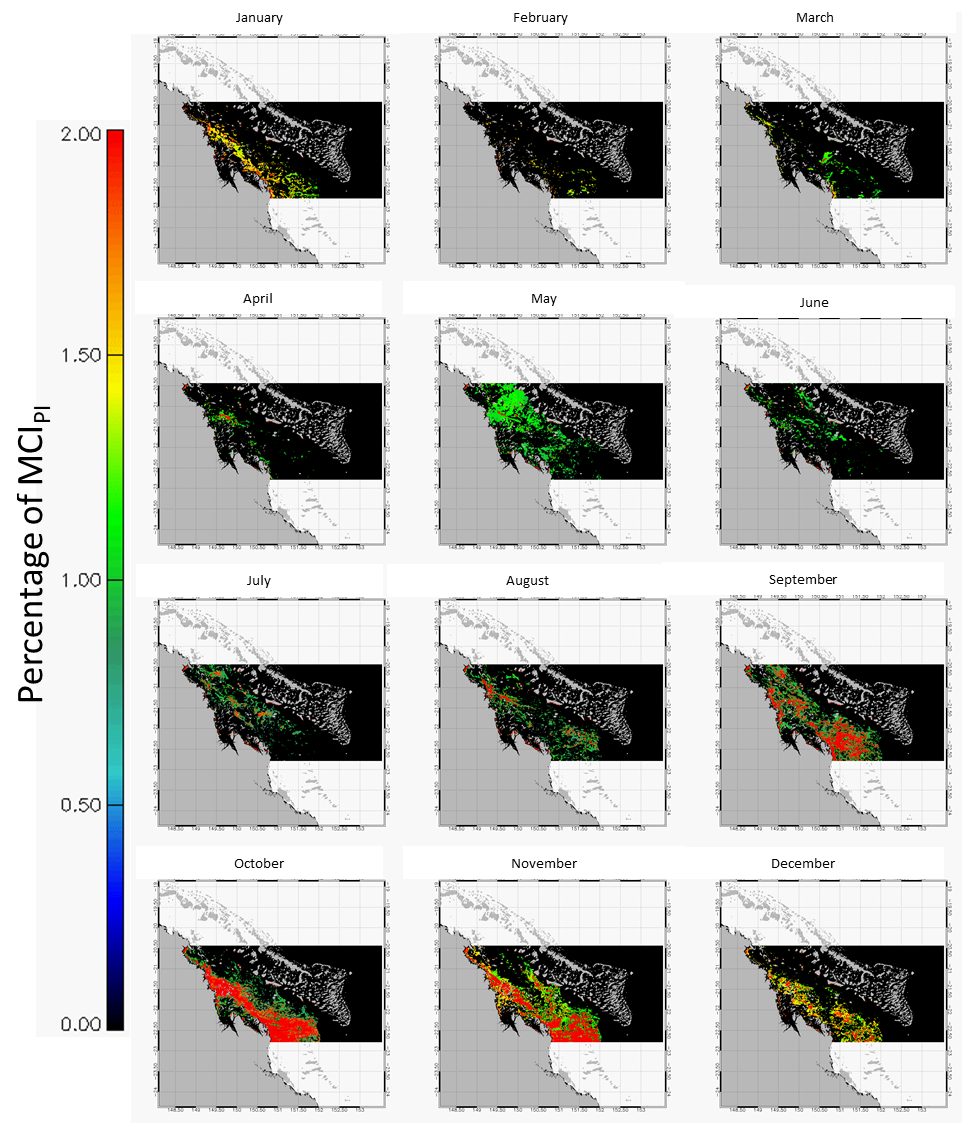

Supplement: S7 Fig — (TIF) [file pone.0208010.s007.tif]
