# Supplementary material for: Phenology of Trichodesmium spp. blooms in the Great Barrier Reef lagoon, Australia, from the ESA-MERIS 10-year mission
Source: PLoS One. 2018 Dec 14;13(12):e0208010. doi: 10.1371/journal.pone.0208010 (PMC6294392; doi:10.1371/journal.pone.0208010)
Supplement: S1 Table — Significant p-values (α = 0.05) are indicated in bold. (DOCX) [file pone.0208010.s008.docx]

| Effect | df^i^ | Chi square^ii^ | *p*-value |
| --- | --- | --- | --- |
| s(SST) | 3.0 | 52.5 | **<0.001** |
| Region (main effect) | 4 | 118.6 | **<0.001** |
| s(Julian Date, by region: Cape York) | 4.9 | 91.3 | **<0.001** |
| s(Julian Date, by region: Cairns) | 8.7 | 20.2 | **0.017** |
| s(Julian Date, by region: Burdekin) | 5.1 | 90.4 | **<0.001** |
| s(Julian Date, by region: Mackay) | 4.1 | 43.6 | **<0.001** |
| s(Julian Date, by region: Fitzroy) | 5.0 | 129.9 | **<0.001** |
| s(Year) | 8.3 | 33.0 | **<0.001** |

* “s()” denotes smoother applied to predictor variable to account for non-linear effect on probability of bloom presence/absence.

^i^ “effective degrees of freedom” are reported for smoothed model terms.

^ii^ test statistic for assessing significance of smoothed model terms. Analogous to “Z-value” for parametric coefficients.
