# Supplementary material for: Phenology of Trichodesmium spp. blooms in the Great Barrier Reef lagoon, Australia, from the ESA-MERIS 10-year mission
Source: PLoS One. 2018 Dec 14;13(12):e0208010. doi: 10.1371/journal.pone.0208010 (PMC6294392; doi:10.1371/journal.pone.0208010)
Supplement: S2 Table — Significant p-values (α = 0.05) are indicated in bold. (DOCX) [file pone.0208010.s009.docx]

| Effect | df^i^ | Chi square^ii^ | *p*-value |
| --- | --- | --- | --- |
| s(SST) | 2.1 | 3.0 | 0.319 |
| Region (main effect) | 4 | 119.2 | **<0.001** |
| s(Julian Date, by region: Cape York) | 2.1 | 9.3 | **0.027** |
| s(Julian Date, by region: Cairns) | 1.0 | 0.5 | 0.503 |
| s(Julian Date, by region: Burdekin) | 1.0 | 1.4 | 0.236 |
| s(Julian Date, by region: Mackay) | 3.4 | 48.9 | **<0.001** |
| s(Julian Date, by region: Fitzroy) | 6.3 | 150.2 | **<0.001** |
| Year | 1 | 5.2 | **0.023** |

* “s()” denotes smoother applied to predictor variable to account for non-linear effect on bloom extent.

^i^ “effective degrees of freedom” are reported for smoothed model terms.

^ii^ test statistic for assessing significance of smoothed model terms. Analogous to “Z-value” for parametric coefficients.
