# Supplementary material for: Correction: Phenology of Trichodesmium spp. blooms in the Great Barrier Reef lagoon, Australia, from the ESA-MERIS 10-year mission
Source: PLoS One. 2019 Mar 5;14(3):e0213559. doi: 10.1371/journal.pone.0213559 (PMC6400371; doi:10.1371/journal.pone.0213559)
Supplement: S1 File — (PDF) [file pone.0213559.s001.pdf]

RESEARCH ARTICLE

Phenology of *Trichodesmium* spp. blooms in the Great Barrier Reef lagoon, Australia, from the ESA-MERIS 10-year mission

David Blondeau-Patissier[1,2¤]*, **Vittorio Ernesto Brando**[3,4], **Christian Lønborg**[5], **Susannah M. Leahy**[6], **Arnold G. Dekker**[4]

1 Research Institute for the Environment and Livelihoods (RIEL), Charles Darwin University, Darwin, Australia, **2** North Australia Marine Research Alliance (NAMRA), Darwin, Australia, **3** National Research Council (CNR), Institute of Atmospheric Sciences and Climate, Rome, Italy, **4** Commonwealth Scientific and Industrial Research Organization (CSIRO), Canberra, Australia, **5** Australian Institute of Marine Science (AIMS), Townsville, Australia, **6** College of Science and Engineering, James Cook University, Cairns, Australia

¤ Current address: Commonwealth Scientific and Industrial Research Organization (CSIRO), Brisbane, Australia
* david.blondeau-patissier@csiro.au

Check for updates

OPEN ACCESS

Citation: Blondeau-Patissier D, Brando VE, Lønborg C, Leahy SM, Dekker AG (2018) Phenology of *Trichodesmium* spp. blooms in the Great Barrier Reef lagoon, Australia, from the ESA-MERIS 10-year mission. PLoS ONE 13(12): e0208010. https://doi.org/10.1371/journal.pone.0208010

Editor: Arga Chandrashekar Anil, CSIR-National Institute of Oceanography, INDIA

Received: April 22, 2018

Accepted: November 9, 2018

Published: December 14, 2018

Copyright: © 2018 Blondeau-Patissier et al. This is an open access article distributed under the terms of the Creative Commons Attribution License, which permits unrestricted use, distribution, and reproduction in any medium, provided the original author and source are credited.

Data Availability Statement: All relevant data are within the paper and its Supporting Information files.

Funding: For this study, DBP was partly supported by a NAMRA post-doctoral fellowship and by CSIRO Oceans and Atmosphere funding. VEB was supported by CNR, the RITMARE flagship Project and the European Union (FP7-427 People Co-funding of Regional, National and International Programmes, GA n. 600407). Collection of the

Abstract

Trichodesmium, a filamentous bloom-forming marine cyanobacterium, plays a key role in the biogeochemistry of oligotrophic ocean regions because of the ability to fix nitrogen. Naturally occurring in the Great Barrier Reef (GBR), the contribution of *Trichodesmium* to the nutrient budget may be of the same order as that entering the system via catchment runoff. However, the cyclicity of *Trichodesmium* in the GBR is poorly understood and sparsely documented because of the lack of sufficient observations. This study provides the first systematic analysis of *Trichodesmium* spatial and temporal occurrences in the GBR over the decade-long MERIS ocean color mission (2002–2012). *Trichodesmium* surface expressions were detected using the Maximum Chlorophyll Index (MCI) applied to MERIS satellite imagery of the GBR lagoonal waters. The MCI performed well (76%), albeit tested on a limited set of images (N = 25) coincident with field measurements. A north (Cape York) to south (Fitzroy) increase in the extent, frequency and timing of the surface expressions characterized the GBR, with surface expressions extending over several hundreds of kilometers. The two southernmost subregions Mackay and Fitzroy accounted for the most (70%) bloom events. The bloom timing of *Trichodesmium* varied from May in the north to November in the south, with wet season conditions less favorable to *Trichodesmium* aggregations. MODIS-Aqua Sea Surface Temperature (SST) datasets, wind speed and field measurements of nutrient concentrations were used in combination with MCI positive instances to assess the blooms' driving factors. Low wind speed (<6 $m.s^{-1}$) and SST > 24˚C were associated with the largest surface aggregations. Generalized additive models (GAM) indicated an increase in bloom occurrences over the 10-year period with seasonal bloom patterns regionally distinct. Interannual variability in SST partially (14%) explained bloom occurrences, and other drivers, such as the subregion and the nutrient budget, likely regulate *Trichodesmium* surface aggregations in the GBR.

nutrient data presented in this study was obtained with support from the Great Barrier Reef Marine Park Authority (GBRMPA), through funding from the Australian Government's Caring for our Country Program and from the Australian Institute of Marine Science.

Competing interests: The authors have declared that no competing interests exist.

Introduction

Cyanobacterium *Trichodesmium* sp. is a marine diazotroph [1] phytoplankter found in all tropical and subtropical oceans [2–4]. In recent years, *Trichodesmium erythraeum*, the species occurring in Australian waters, has also been reported in the Mediterranean Sea [5] in late summer-early autumn and in UK coastal waters during winter [6]. *Trichodesmium* is known for forming extensive algal blooms that can cover hundreds of kilometers of the ocean surface. Aggregations have sometimes been so large that the sailors of the HMS Endeavor mistakenly identified a new shoal when the vessel sailed through the Torres Strait (North of Cape York, Australia) in August 1770. These persistent surface expressions occur toward the end of the algal bloom phase [7] and can last several days until the bloom subsides. Composed of senescent cells that are darker than healthy cells due to chlorosis [8], these surface matts increase the water-leaving radiance signal in the red-near-infrared (NIR) spectral region (680–750 nm), also called "red edge" [9], allowing their detection from satellite sensors for the past 30 years [10–12].

Blooms of *Trichodesmium* colonies likely play key roles in the ecosystem because of their ability to fix atmospheric nitrogen [13, 14], thereby contributing to new nitrogen inputs in oligotrophic waters [15–18]. *Trichodesmium* colonies also contribute to the phosphorus budget by the uptake of phosphorus for growth [19, 20], in addition to providing substrate and shelter to various organisms ranging from bacteria to crustacean larvae [21]. In the oligotrophic waters of the Great Barrier Reef (GBR), with chlorophyll-a concentrations that range from ~0.2 to 0.8 $\mu g.L^{-1}$ (e.g., S1 Fig; [22]), nitrogen fixation by *Trichodesmium* blooms may be a major source of new nitrogen, particularly in the offshore parts of the shelf [19, 23].

However, because of the lack of systematic studies of *Trichodesmium* bloom events in the GBR, the understanding of their temporal and spatial dynamics within the system remains limited [4]. Based on field observations or satellite datasets, only a few studies have described the climatology of *Trichodesmium* in this region. The first study (1933) provided the seasonal abundance of *Trichodesmium* off the Low Islands (North of Cairns; Fig 1) over one annual cycle [24]. A major burst (~180 $trichomes.L^{-1}$) occurred in August and was followed by another, although lower (~145 $trichomes.L^{-1}$) but more sustained, peak in November-December. Four decades later, taxonomic counts of *Trichodesmium* at three stations off Townsville visited in 1976 and 1977 also reported the highest number of cells in August, December and March [25] (Fig 1). A 1992 study reported that *Trichodesmium* surface aggregations occurred mostly between August and February, but the spatio-temporal distributions of the events were uncertain [14]. More recent satellite-based studies have reported on *Trichodesmium* blooms in the western Pacific [26, 27], the north Pacific subtropical gyre [28] and in the Cairns subregion [29]. In the latter, the phenology of *Trichodesmium* events during the ESA-MERIS sensor's mission from 2002 to 2012 occurred predominantly in August.

Thus, the complete spatial and seasonal occurrence of *Trichodesmium* in the GBR as a whole remains unresolved until this study, because to our knowledge, no previous research on the climatologic distribution of *Trichodesmium* has systematically analyzed these events over a decade-long period. This study builds on our previous research for the Cairns subregion [29] and extends this analysis to the whole of the GBR spanning 15˚ of latitude (9.5˚ to 24.5˚ S; Fig 1). Using 10 years of MERIS Reduced Resolution (RR) satellite data and the Maximum Chlorophyll Index (MCI) [30], we provide new insights into the spatial distribution and decade-long temporal dynamics of *Trichodesmium* blooms in the GBR region. By comparing time-series of MCI-positive instances along the GBR lagoonal waters, we identify the spatial and seasonal patterns of *Trichodesmium* surface expressions across the GBR subregions. Complemented by datasets of remotely sensed sea surface temperature (SST), wind speed and *in situ*

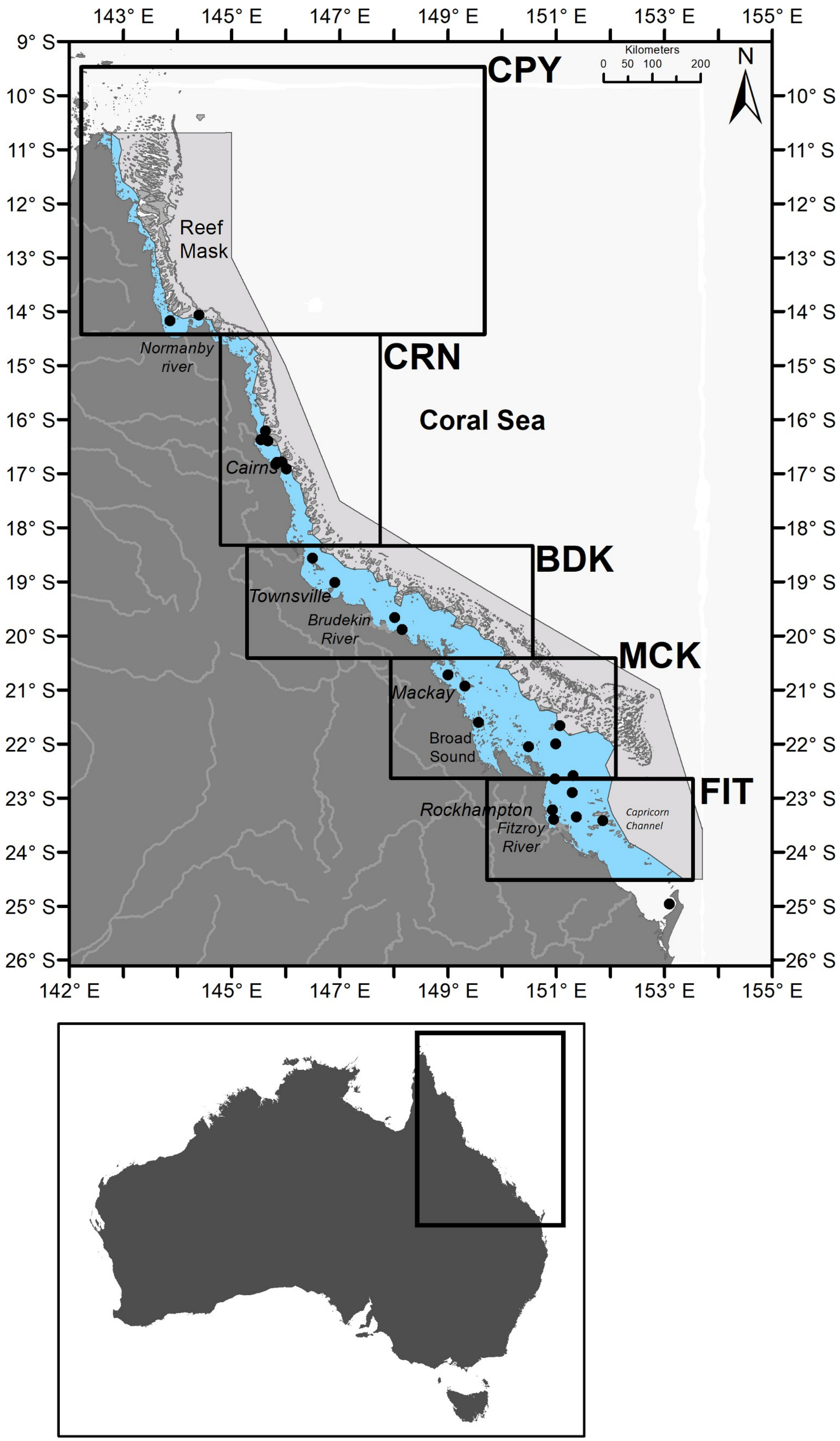
143° E
145° E
147° E
149° E
151° E
153° E
155° E
9° S
10° S
11° S
12° S
13° S
14° S
15° S
16° S
17° S
18° S
19° S
20° S
21° S
22° S
23° S
24° S
25° S
26° S
142° E
CPY
Kilometers
0 50 100 200
N
Reef Mask
Normanby river
CRN
Coral Sea
Cairns
BDK
Townsville
Brudekin River
MCK
Mackay
Broad Sound
FIT
Rockhampton
Fitzroy River
Capricorn Channel

Fig 1. Map of the study region including major cities, rivers, the masking of the reef matrix and the geographic boundaries of the five subregions for this study. Light blue shading indicates the reef lagoon area that was the focus of this study. Black circles indicate the locations at which validation samples were taken.

https://doi.org/10.1371/journal.pone.0208010.g001

nutrients, we assess the primary environmental factors associated with *Trichodesmium* surface expressions within the GBR.

Materials and methods

No specific permissions were required for these locations and research activities. The Australian Institute of Marine Science provided the nutrient datasets. No endangered or protected species were involved in this work.

The study region: Seasonal and hydrological aspects

The GBR has an area of ~350,000 km^2, with 10% covered by coral reefs (>3,000 individual reefs) [31]. This study focuses on the GBR lagoon, the water body located between the shoreline and the coral reef matrix (Fig 1). The width of the lagoon varies from up to 250 km in the southern GBR to less than 25 km north of 18˚ S, as the reef matrix is closer to shore there (Fig 1).

The GBR has a monsoonal climate with most rainfall occurring during the wet season (summer, November to April, >1,500 mm/year), resulting in episodic large river runoffs that may decrease the lagoonal water salinity (<< 33 ppt) and increase turbidity (Secchi disk depth < 1 m; [32]) in the near-shore lagoon [31, 33]. These conditions are observed close to large river catchments, such as the Fitzroy River or the Burdekin River, or in shallow, tidal embayments, e.g., Broad Sound (Fig 1). Rainfall patterns and intensity are also influenced by the El Niño and La Niña climate phases, with El Niño summers characterized by higher than average SSTs, low wind speeds and clear skies, whereas La Niña summers are characterized by lower than average SSTs, higher wind speeds, and more cloudy conditions [34]. During the dry season months (May-October, <300 mm/year), southeast trade winds dominate. Wind strength eases in November-December and changes direction, allowing the intrusion of clearer water masses from the Coral Sea into the central and southern sections of the GBR.

For this study, the GBR was divided into five subregions (Fig 1), geographically defined as per the Natural Resource Management (NRM) areas. Typically, NRMs are used as reporting regions in the GBR report cards and Marine Monitoring Program (MMP). The water bodies vary in width from north to south. Cape York (CPY; Fig 1) is the subregion closest to the reef matrix. Most of the land in this subregion is undeveloped, with extensive land areas dedicated to nature conservation [35]; thus, the water quality is considered relatively pristine [36]. The Normanby is the major river and flows into Princess Charlotte Bay. Most of the coastal catchments in CPY are subject to heavy tropical rainfall. Although composed of small rivers, the CPY river catchments have an annual discharge equal to one-fourth of the entire GBR [31]. The Cairns subregion (CRN; Fig 1) is characterized by wet tropical rainforest and cleared land with agriculture being the major land-use. The primary rivers influencing the CRN are the Mossman, the Daintree, the Johnstone and the Tully rivers. In the Burdekin subregion (BDK; Fig 1), agriculture (cattle grazing in particular) is the main (90%) land-use activity [35]. The BDK is one of the largest (130,000 km^2) catchments in the GBR, and its main river, the Burdekin, can generate extensive river plumes during the wet season [33, 37]. The Mackay subregion (MCK; Fig 1) is the farthest from the reef matrix (the GBR lagoon is the widest there) and hosts most of the sugarcane industry. The Fitzroy subregion (FIT; Fig 1) experiences a semi-arid to subtropical climate, and the land-use is characterized by cattle grazing [35]. The Fitzroy

River is the largest river system and catchment (140,000 km^2) discharging into the GBR [38, 39].

Nutrient and metadata dataset from *in situ* sampling

In situ sampling of physical and chemical variables was conducted in each of the five subregions over the period 2002–2013. Data are summarized in S1 Fig. All water samples were collected with Niskin bottles, salinity and temperature were determined with a CTD SeaBird 911, and chlorophyll-a concentrations were measured fluorometrically using a Turner Designs 10 AU fluorometer. Further description of the methods used for this analysis can be found in [40, 41].

Satellite image processing

During its mission, the MERIS ocean color sensor onboard the Envisat satellite provided reduced resolution (RR; 1.2 Km) imagery acquisitions of the GBR every two days on average. MERIS full resolution (FR; 300 m) imagery was intermittently acquired over Australia. Because the aim of this study was to use the entire MERIS mission, only RR imagery was used for this analysis. A total of 4,681 MERIS RR scenes from the third reprocessing, covering parts of the GBR between April 29, 2002, and April 4, 2012 (at ~10 a.m. local time; UTC+10), were downloaded from the Optical Data processor of the European Space Agency (ODESA) provided by ACRI-ST (www.odesa-info.eu/). Subsets bounded to the five geographic subregions of interest were created, resulting in a total of 7,234 MERIS subscenes (S2 Fig). Land mass, cloud and sun glint-contaminated pixels were masked using the MERIS Level 1 quality flags.

The MERIS Maximum Chlorophyll Index (MCI) [42] was used for the detection and mapping of surface algal bloom expressions. The MCI algorithm is primarily designed for the detection of algal blooms with very high Chl-a concentrations globally (>30 $\mu g.L^{-1}$) [30, 42], but such Chl-a concentrations are very high in comparison to the concentration ranges found in the waters of our study area: the GBR lagoonal waters are typically oligotrophic (Chl < 1 $\mu g.L^{-1}$) (e.g., [22, 43], S1 Fig), with higher Chl-a concentrations measured sporadically during the wet season [40, 44]. Previous studies show that MCI can be used to detect surface blooms of slick-forming algal species such as *Sargassum* [45–47] in the Gulf of Mexico and Atlantic Ocean, in addition to *Trichodesmium* [10, 29, 48, 49]. Computed from three MERIS bands in the near-infrared, namely, 681, 709, and 753 nm [50, 51], the MCI was defined as follows:

$$\mathrm{MCI} = L709 - k \times \left[L681 + (L753 - L681) \times \frac{709 - 681}{753 - 681} \right] \tag{1}$$

where L_λ are level 1 TOA radiances at wavelengths λ using MERIS bands 10 (753 nm), 9 (709 nm) and 8 (681 nm), and *k* is a cloud factor set at the value of 1.005 and used to correct the influence of thin clouds.

The MCI algorithm was applied to each Level 1 scene using the VISAT BEAM software toolbox (now replaced by the Sentinel Application Platform (SNAP)) and the default cloud correction factor of 1.005 to reduce the effect of thin clouds [52]. Processing the images to Level 2 included an atmospheric correction step, which might either result in flagging *Trichodesmium* sp. pixels as erroneous or as a saturated product over brighter bloom areas because radiance values for these pixels would likely be outside the expected range of the MERIS atmospheric correction [42, 53]. MCI radiances typically vary between -3 and ~15 $mW^{-2}.sr^{-1}.nm^{-1}$, with positive values above an MCI background level indicating phytoplankton-laden pixels. The MCI product does not have refined flags; thus, quality control was insured by computing the MCI for pixels with Level 1 top of atmosphere (TOA) radiance at 865 nm as <15 $mW^{-2}.sr^{-1}.nm^{-1}$ [30], which

further filtered pixels contaminated by land, high sun glint, haze or thick clouds. Disadvantages of the MCI include its sensitivity to submerged reefs [48, 54], because coral reefs contain zooxanthellae that may trigger a positive MCI response [42]. A mask was applied to separate the GBR lagoon from the Coral Sea, thereby covering the entire reef matrix and avoiding the inclusion of possible false positives from the coral reef signals (Fig 1). The total counts of positive MCI values for each scene, hereafter called MCI positive instances and noted as MCI_{PI}, were defined as:

$$MCI_{PI} = \sum_{i=0}^{n} \mathrm{MCI} > (\mathrm{t} - \mathrm{b}) \quad (2)$$

where *t* is the MCI threshold value, *b* is the background MCI value and *n* is the number of valid MCI pixels (i.e., "valid" refers to pixels for which a meaningful value is obtained not flagged for cloud, glint or any other L1 quality flag).

The value of *b* is always negative, and as a result, the term (t-b) is always positive. The threshold *t* and median ocean background MCI values *b* of +0.4 $mW^{-2}.sr^{-1}.nm^{-1}$ and -0.4 $mW^{-2}.sr^{-1}.nm^{-1}$, respectively, were selected based on Gower et al. [48, 55]. However, we found that the sun illumination effects and the sensor-sun geometry were highly variable across regions covering 14˚ of latitude, and the *Gower et al*. background value of -0.4 $mW^{-2}.sr^{-1}.nm^{-1}$ was deemed not representative for our study region. For this study, *t* was a constant set at 0 $mW^{-2}.sr^{-1}.nm^{-1}$, and *b* was computed for each scene by taking the mean MCI value of non-bloom-contaminated pixels in the area surrounding the blooms. The component (*t-b*) varied in accordance with the viewing or sun angle.

Remotely sensed datasets of sea surface temperatures (SST) were used to support our analysis and assess the relationship between SST and *Trichodesmium* occurrences in the GBR lagoon. SST datacubes were composed of the daily NASA-MODIS Aqua skin temperature datasets for the period spanning the MERIS acquisition dates starting from July 4, 2002 to April 7, 2012. A box delineating the boundaries of each subregion and the same masks used for the MCI_{PI} data covering the land, the reef matrix and the Coral Sea were also applied to the SST. SST daily average values were extracted for each subregion for the lagoon waters only. Across the entire time-series for all regions, SST varied from 17.5˚C to 29.9˚C, with an overall median of 23.5˚C.

Algorithm validation

The performance of MCI_{PI} at retrieving *Trichodesmium* bloom surface expressions was assessed using field observations collated from four sources: the GBR-wide marine monitoring program (MMP), the GBRMPA database, CSIRO field campaigns and those listed in [56]. These records provided only qualitative observations (*i.e*., absence, presence) of *Trichodesmium* bloom surface expressions. The extent of the surface aggregations was not available, because these records were not collected explicitly for satellite validation purposes. Only the location (latitude, longitude), datum and the qualitative observation (absence, presence) were indicated. Records labeled as "present" between April 2002 and June 2009 were used (N = 432). For the purpose of this validation exercise, the search for the listed *Trichodesmium* events was conducted following McKinna et al [56]: independently of the sea-truth observation time, MERIS images within ±2 days of the observation date were used. The location of the field observation was used as the center pixel of a 10 x 10 pixel box (equating to ~100 km^2). The maximum MCI_{PI} value within that pixel box was extracted and used to determine absence or presence of *Trichodesmium* aggregations. When several images showed positive results for a single field observation, only the image the closest in time and date to the observation was considered. A coral reef mask was applied to each selected scene.

Wind speed datasets

Daily average wind speed was estimated in each of the five subregions from the eReefs CSIRO Environmental Modelling Data Node (www.ereefs.org.au/). The GBR 4-km hydrodynamic model was used to estimate the daily eastward and northward wind components from which the wind speed and direction were computed. The components were modeled at 10 a.m. local time to match the overpass time of the MERIS satellite sensor. Only daily wind speed data from September 1, 2010, onward were available, which limited the dataset to a total of 581 days (*i.e.*, September 1, 2010-April 4, 2012).

Data analysis and generalized additive model (GAM) selection

To analyze the seasonal patterns of *Trichodesmium* blooms in the GBR, MCI_{PI} were extracted from all MERIS scenes for the five subregions (Fig 2). Only daily subscenes with a minimum of 10 MCI_{PI} were counted as containing bloom events and used for the subsequent analysis, and the value of *b* (Eq 2) was computed on at least 50 valid pixels. Using the R package "wq" for water quality data analysis [57], daily MCI_{PI} were aggregated into monthly means to construct a 121-month time-series for the five subregions, and decadal trend models were computed using semiparametric smoothing techniques. An overall average of 13 MERIS scenes per month was available for monthly aggregations for a specific subregion. MERIS scenes with missing pixels were highly seasonal and occurred mostly during the wet season, i.e. January-March, due to cloud cover. The southern regions of MCK (2%) and FIT (4%) were the least affected by missing data, whereas CRN (33%) was the most affected (S2 Fig).

Two generalized additive models (GAMs) were built to model the influence of predictors (SST, time of year, interannual variation, and region) on the occurrence of *Trichodesmium* blooms, based on our response variable (MCI_{PI}). Wind was not used as a predictor, because the wind dataset was limited to only two years of the study period. Models were based on daily data collected between July 4, 2002, and April 4, 2012. Days for which $< 20\%$ of a scene was visible (i.e., $\geq 80\%$ cloud cover) were excluded from the analysis. The resulting dataset was zero-inflated (45%) and was analyzed using the two-step hurdle approach: (1) explain presence/absence, and (2) when present, explain extent [58].

Firstly, to assess the probability of a *Trichodesmium* bloom occurring, a GAM was fitted to the binary data (bloom or no bloom, hereafter called Bernoulli) using the *mgcv* package in R and a binomial link function. A GAM approach was used because several key predictor variables were considered likely to have non-linear effects: SST, time of year (or seasonality, represented by Julian Date) and interannual effects (represented by year). To test for regional differences in seasonality, the factor "region" was included in the model as both a main effect and as a varying coefficient of Julian Date (similar to an interaction effect). Secondly, a beta GAM was used to assess the variables influencing the extent of *Trichodesmium* blooms, within the subset of data in which blooms had been observed to occur. The response variable "bloom extent" was represented as the number of bloom pixels divided by the number of valid pixels in each image. This approach controlled for differences in the number of valid pixels in each image (e.g., non-cloud pixels) and for differences in the size of each region. The resulting variable was a proportion that did not include any zeroes or ones and was therefore ideally suited to a beta distribution. The response variable was cube root transformed to increase the spread of data and improve model dispersion, while staying within the constraints of the beta distribution. The beta GAM was fitted using a theta of 30 and the identity link function. Smoothers were applied to both SST and time of year data for the Bernoulli and beta GAMs.

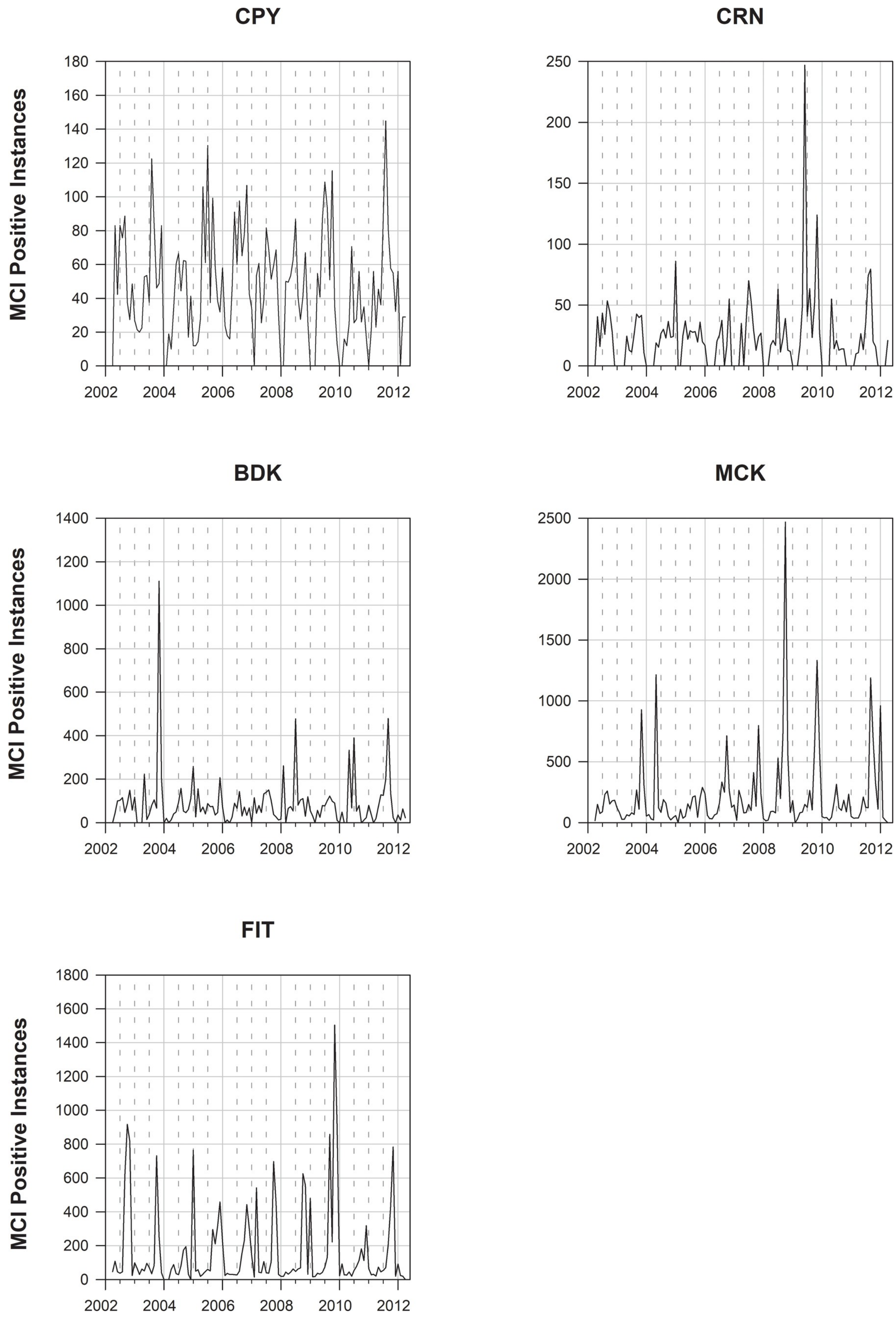
CPY
CRN
BDK
MCK
FIT
MCI Positive Instances
0 20 40 60 80 100 120 140 160 180
0 50 100 150 200 250
0 200 400 600 800 1000 1200 1400
0 500 1000 1500 2000 2500
0 200 400 600 800 1000 1200 1400 1600 1800
2002 2004 2006 2008 2010 2012

Fig 2. Time series of monthly averaged MCIPI for the five subregions (ordered from north to south). Vertical dashed lines represent 6-month periods for all plots.

https://doi.org/10.1371/journal.pone.0208010.g002

Results

A summary of the physical and chemical variables in the surface waters of each subregion for the period 2002–2013 is presented in S1 Fig. Distinct differences were found in averaged key environmental parameters collected in the five subregions, with the largest spatial variations for temperature, particulate organic carbon (POC), nitrogen (PN), phosphate (PP), dissolved inorganic nitrogen (DIN) and organic phosphate (DOP), total dissolved nitrogen (TDN) and phosphate (TDP). Most of the nine variables showed slight north to south gradients across the five subregions: temperature decreased from CPY to FIT by up to 3.5˚C associated with the change in latitude (tropical to subtropical), whereas Chl-a showed little change (s.d.~0.2 $\mu g.L^{-1}$) across the subregions. Some of the nutrients (e.g., POC, DIN, and TDP), with the exception of PP, showed minor increases in concentrations across the subregions on a north to south gradient. DIN and PN showed a very minor increase from CPY to BDK, and notably, DOP showed a >80% increase from CPY to FIT. This latitudinal gradient in nitrogen and phosphorus is consistent with recent findings on GBR water quality [40, 59], with greater reported loads of DIN and PN in the southern GBR.

McKinna et al. used a set of 13 NASA-MODIS high-resolution (250 m) images coincident with *Trichodesmium* events observed in the central GBR (16˚-23˚ S) to validate a binary classification algorithm [56]. The events used for the satellite retrieval assessment in their study were recorded in January 2005, April and October 2007, July, September, October, and November 2008 and February and June 2009. The algorithm retrieval was robust (85% success), albeit tested on a limited number of scenes (N = 13). Our validation exercise used 25 field observations with matching MERIS images (Table 1). Observations were reported in all months except March and December, with more than half (52%) recorded between September and November. A total of 19 these records had positive MCI_{PI}, equivalent to 76% success. A total of nine MERIS scenes retrieved 13 of the sightings listed in *McKinna et al.* [56]. The distribution of the site locations is shown in Fig 1.

Table 1. $MCIP_{PI}$ validation results. Annual distribution of *Trichodesmium* field records noted as "present" with matching MERIS images (N = 25): columns 1–2: number of recorded field observations for each month; col. 3: corresponding frequency of these observations; cols. 4–5: MERIS retrieval statistics showing whether recorded field observations were retrieved by a MERIS image or not in the matching image pool.

Month	N	%	Retrieved	Not retrieved
**January**	1	4	0	1
**February**	2	8	2	0
**March**	—	—	—	—
**April**	3	12	0	3
**May**	1	4	0	1
**June**	2	8	2	0
**July**	2	8	2	0
**August**	1	4	1	0
**September**	5	20	5	0
**October**	5	20	5	0
**November**	3	12	2	1
**December**	—	—	—	—
**Total**	25	100	19	6

https://doi.org/10.1371/journal.pone.0208010.t001

The number of MERIS images varied across subregions, with the lowest number of scenes for CRN (N = 1,117) and FIT with the most (N = 1,788) (Table 2). A strong seasonality in satellite data availability was apparent, with the number of valid observations sharply decreasing during the wet season months because of increased cloud cover (S2 Fig) [60]. The magnitude of this seasonal decline decreased from CPY in the wet tropics to the subtropical region of FIT in which the wet season cloud cover was less pronounced. The monthly time-series of MCI background value, *b*, for each of the five regions is shown in S3 Fig. The difference in sun elevation and satellite observation angle variability across the 14° of latitude and the seasons resulted in a 25% difference in mean MCI background value between the northernmost (CPY; -0.8 $mW^{-2}.sr^{-1}.nm^{-1}$) and the southernmost (FIT; -0.6 $mW^{-2}.sr^{-1}.nm^{-1}$) regions. Highest MCI background values were observed around June (early dry season) for all regions.

The multi-annual times-series of monthly averaged MCI_{PI} for the five subregions are shown in Fig 2. The two southernmost subregions MCK and FIT accounted for >60% of all MCI_{PI} for the GBR (Fig 2). Identical patterns were observed when these time-series were presented as ratios between daily MCI_{PI} and the corresponding number of satellite observations for each region (data not presented). A general increase in MCI_{PI} occurred from north to south, with CPY and CRN with the lowest (N = 272) overall number of monthly maximum counts (Fig 2) in comparison with the southernmost regions MCK and FIT for which monthly counts reached up to >2,000. Years with the most MCI_{PI} varied across subregions but all occurred from 2007 onward with the exception of BDK (Fig 2; Table 3). The decadal seasonal signals (Fig 3) and their phenology (Fig 2) explicitly demonstrated that the peak of surface expressions captured in the imagery had a north to south gradient. The difference in algal bloom sizes also had a north to south increasing gradient in algal bloom area coverage, which was aligned with the overall increase in nutrients from northern to southern regions (e.g., [33], S1 Fig). The largest bloom events found in each of the five subregions are shown in Fig 4. In these instances, surface expressions were estimated to extend over 20,000 km^2 with as many as 10,500 MCI_{PI} in a single scene (e.g., MCK). Although blooms mostly occurred in July-August in CPY and CRN [29] in the north, a progressive shift occurred toward September in MCK and October-November in FIT (Fig 5). When all months were considered over the 10-year period, the largest number of bloom events occurred in 2006 (CPY), 2008 (MCK), and 2009 (CRN, FIT) (Table 3**)**. The years 2009–2010 were moderate El Niño years and therefore characterized by limited rainfall. The year 2011 was a wet year characterized by large river discharges in the BDK in particular and associated low MCI_{PI} counts. The *Trichodesmium* bloom of October 2008 in MCK is also reported in Fig 8 in McKinna [10], whereas the large bloom events of August 2011, mostly covering the northern GBR, are well known and featured in NASA's Earth Observatory Image Of the Day for this period (e.g., https://earthobservatory.nasa.gov/IOTD/).

The occurrence and extent of *Trichodesmium* blooms were analyzed following the two-step hurdle approach to: (1) explain the occurrence of blooms and (2) where present, assess the variables influencing the extent of *Trichodesmium* blooms. The Bernoulli GAM modeling the

Table 2. Statistics for the MERIS imagery dataset used in this study. Number of images for each subregion with $MCI_{PI} > 10$ for the five regions and percentage these images account for as a function of the total number of images.

	CPY	CRN	BDK	MCK	FIT
**Size x10^3 km^2**	30	36	42	54	40
**N_{images} Total**	1426	1117	1284	1619	1788
**N_{images} MCI_{PI}**	489	200	416	740	771
**%**	34	18	32	46	43

https://doi.org/10.1371/journal.pone.0208010.t002

Table 3. Monthly and yearly time-series statistics: Maximum MCI_{PI} computed for each subregion when monthly and yearly aggregations were considered; dates are indicated between brackets. Decadal MCI_{PI} (last column) and associated percentage of the total (all regions considered) were based on monthly aggregations over the 10-year period.

Region	Monthly	Yearly	Σ MCI_{PI} for 2002–2012 period
**CPY**	145 (August 2011)	703 (2006)	5,600 (9%)
**CRN**	247 (June 2009)	645 (2009)	2,752 (4%)
**BDK**	1,111 (Nov 2003)	1,956 (2003)	10,484 (16%)
**MCK**	2,469 (Oct 2008)	4,886 (2008)	25,913 (40%)
**fitted**	1,504 (Nov 2009)	4,315 (2009)	20,116 (31%)
**Total**			64,865 (100%)

https://doi.org/10.1371/journal.pone.0208010.t003

probability of a *Trichodesmium* bloom occurrence was fitted to the Bernoulli model assumptions (normality of residuals and homoscedasticity) and displayed an R^2 of 14% (N = 3,732). All of the variables included in the model had significant effects on the probability of a *Trichodesmium* bloom occurring (S1 Table). The effect of SST was strongly significant and non-linear, with a steep increase in probability of a bloom with increasing temperature up to approximately 24˚C and a near-plateau in probability of a bloom at temperatures above 24˚C (not shown). From the GAM analysis, the probability of a bloom occurrence peaked near the 200th day of the year (mid-July) in the northernmost regions and progressively later in the year at more southerly regions (S1 Table), corroborating the graphical analyses of the monthly patterns in MCI_{PI} (Fig 3).

The beta GAM modeling the extent of *Trichodesmium* blooms was fitted to the beta model assumptions [61]. The prediction model was not over-dispersed (<1) and displayed an R^2 of 18.5% (N = 2,106) (S2 Table). A non-linear effect of SST was observed, with a statistically non-significant peak in bloom extents at 23 to 24˚C (p = 0.319). The largest measured influence on the extent of *Trichodesmium* blooms was subregion, with a highly significant regional main effect, in addition to a changing pattern in seasonal effects by region (S2 Table). Year had a weakly positive effect, with increasing extent of *Trichodesmium* blooms over the 10 year study period observed in all regions (Fig 6).

Overall, surface expressions of *Trichodesmium* blooms occurred more frequently from approximately July-August at northerly latitudes to November-December at more southerly latitudes. In CPY, surface expressions occurred for the months of August-September, with a second peak in November (S4 Fig). These later bloom events were mostly located in or close to Princess Charlotte Bay (Fig 1). In CRN, surface expressions tended to appear between June and September (S5 Fig) [29]. In BDK, the surface bloom seasonal activity (S6 Fig) was similar to that in CRN, although blooms could last until December and tended to occur near the mouth of the Proserpine River. In contrast to CPY and CRN, the spatial distribution of *Trichodesmium* surface expressions in BDK was widespread across the entire subregion and showed a higher frequency in pixel counts (S5 Fig). MCK and FIT had the greatest frequency and extent of surface blooms on the GBR; peaks in these regions occurred from September to November (S7 Fig): surface expressions in these subregions were widespread both along the coastline and across the lagoon. FIT had the highest percentage of bloom coverage over the lagoon for all subregions considered, with bloom spatial extents that covered up to 80% of the region in October. MCK had the second largest coverage (>60%).

The average daily wind speed corresponding to events with MCI_{PI} larger than 100 varied between 1.6 and >8 $m.s^{-1}$ (N = 313) across all regions (Tables 4 and 5). Although no strong correlation was found between the two parameters, MCI_{PI} increased when wind speeds dropped below 6 $m.s^{-1}$. The average wind speed was 1.66 $m.s^{-1}$ lower during large bloom

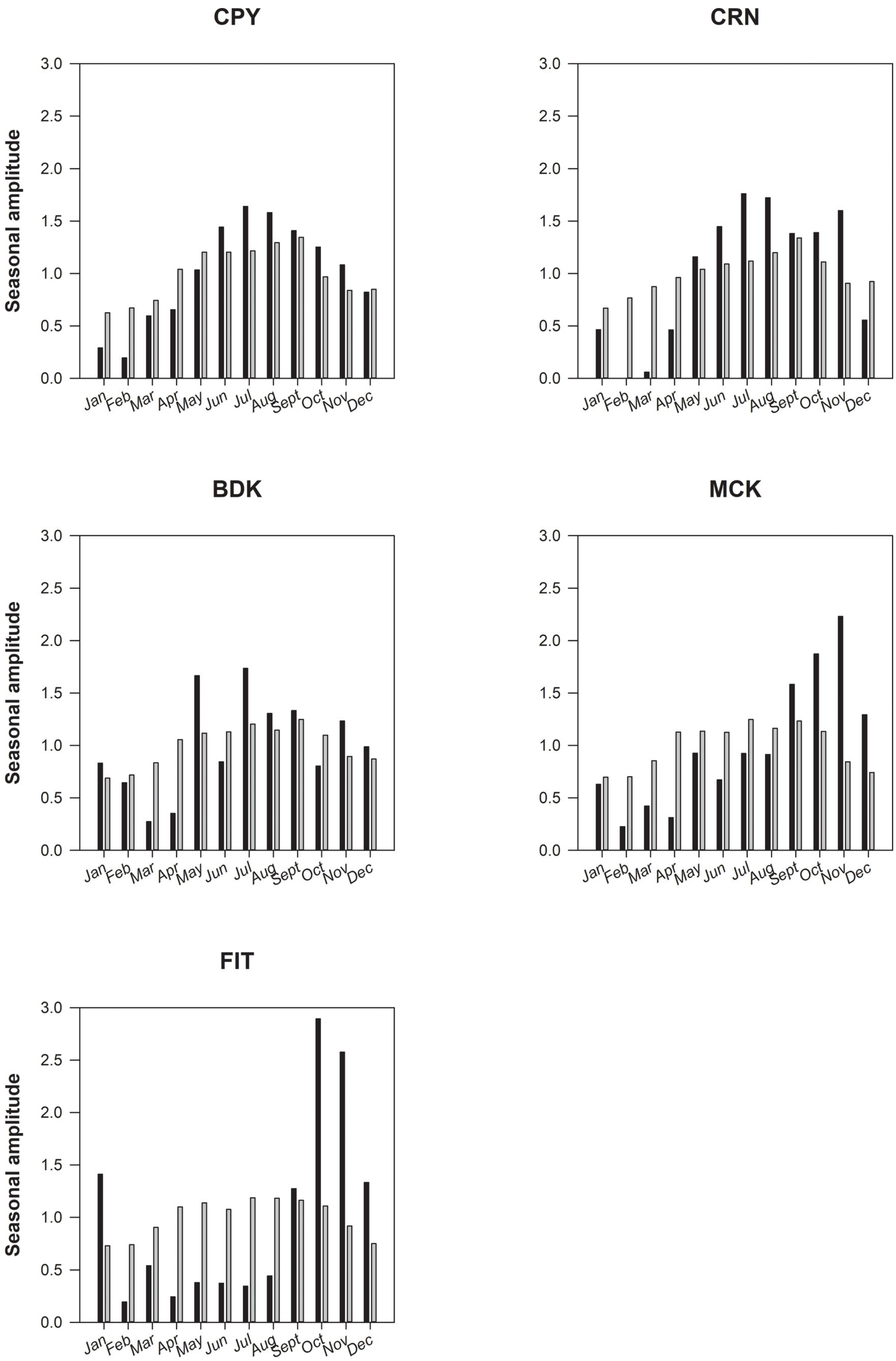
CPY
CRN
BDK
MCK
FIT
Seasonal amplitude
0.0
0.5
1.0
1.5
2.0
2.5
3.0
Jan Feb Mar Apr May Jun Jul Aug Sept Oct Nov Dec

Fig 3. Decadal seasonal amplitudes of MCIPI (black) and valid satellite observations (gray) for the five subregions. The sum of all the seasonal amplitudes over the course of a year for each subregion is equal to 12. The strength of the seasonal signal explains the differences in bar sizes.

https://doi.org/10.1371/journal.pone.0208010.g003

events (>2,000 MCI_{PI}; 5.27 $m.s^{-1}$) than that during smaller (<500; 6.93 $m.s^{-1}$) blooms (Tables 4 and 5). The wind direction recorded for these aggregations, independently of their size, was largely (>80%) from the east-southeast, which indicated that the floating aggregations were pushed toward the shore.

Discussion

On a global scale, the onset and termination phases of *Trichodesmium* blooms depend on many environmental factors [62, 63], which include the combination of optimal salinity [64] and light [65, 66] conditions, nutrient (iron and/or phosphorus) availability [67], the lack of predation (e.g., copepod grazing or viral lysis) [68, 69], water column stability [70] and temperature [71, 72]. In the Indian Ocean (off Zanzibar, 6˚ S), regular *Trichodesmium* aggregations are likely influenced by the warming and shallowness of the mixed layer in the summer, and the blooms are spatially sustained by iron supply carried eastward from the upwelling region south of Madagascar [73]. In the Red Sea (20˚-25˚ N), *Trichodesmium* peaked in July [74]. From our results, seasonal and spatial patterns of *Trichodesmium* aggregations were

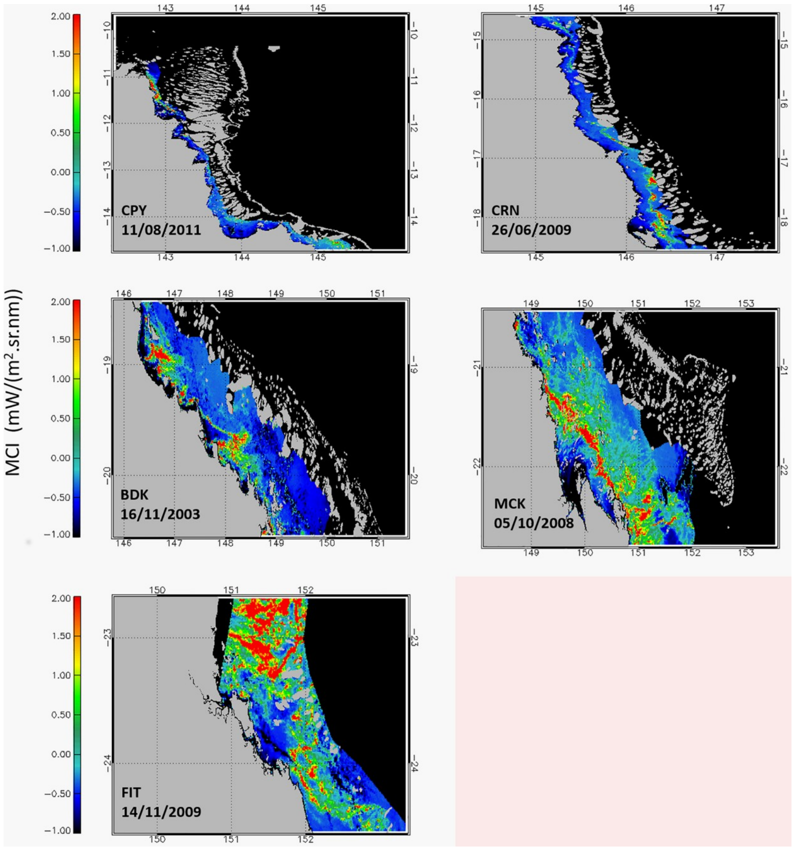

Fig 4. MERIS scenes featuring the largest surface expressions (in size and number of MCI_{PI}) occurring in the lagoonal waters of each subregion between April 2002 and April 2012. The reef matrix and the land are masked in gray.

https://doi.org/10.1371/journal.pone.0208010.g004

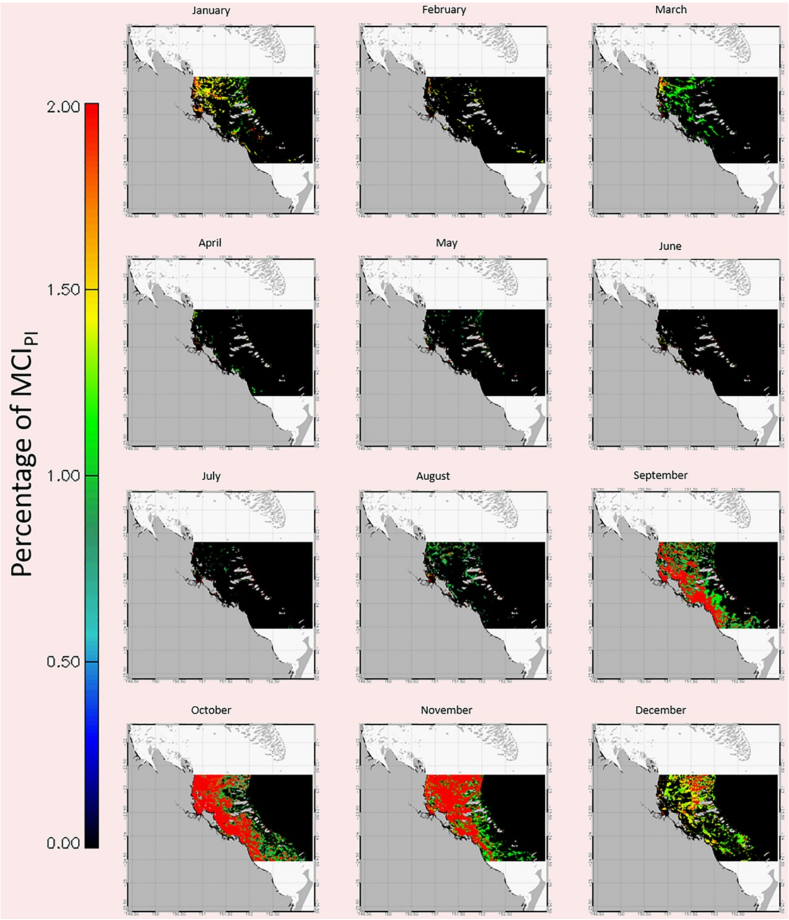

Fig 5. Monthly climatology of surface bloom occurrences (%MCIPI) for FIT over the period April 2002-April 2012. See S4–S7 Figs for the four other subregions.

https://doi.org/10.1371/journal.pone.0208010.g005

evident for the GBR. Previous studies identified the possible environmental triggers of these highly seasonal surface blooms as a combination of low wind speed, nutrient availability (required for a large bloom to occur and persist [75]) and a marginal role played by SST. Yet, it has also been shown that *Trichodesmium* abundance and seawater temperature may not have clear relationships [63, 76, 77], in contrast to other studies in which temperature plays a key role [15, 62, 72].

Strong La Niña phases occurred in 2007–2008 and 2010–2011, while they were moderate in 2011–2012 and weak in 2005–06, 2008–2009. A previous study from Westberry and Siegel [2] used SeaWiFS satellite reflectances to map the spatio-temporal distributions of *Trichodesmium* events at a global scale over a six-year period (1998–2003). The GBR was masked out because their study solely focused on open ocean regions (see Westberry and Siegel [2]). The influence of SOI on *Trichodesmium* blooms was also examined, and the authors reported a correlation of ~25% with La Niña events corresponding to larger and more frequent events. The authors noted that this correlation was particularly evident at the start of their time-series (1998–2000) because a strong El Niño–La Niña transition period occurred. A similar transition might be observed over the period 2009–2011 in our study region, which might partly explain the increased frequency of bloom events noted above. The 2011 La Niña year was characterized by a decrease in salinity, increased nutrients and Chl-a and warmed sea temperatures along the

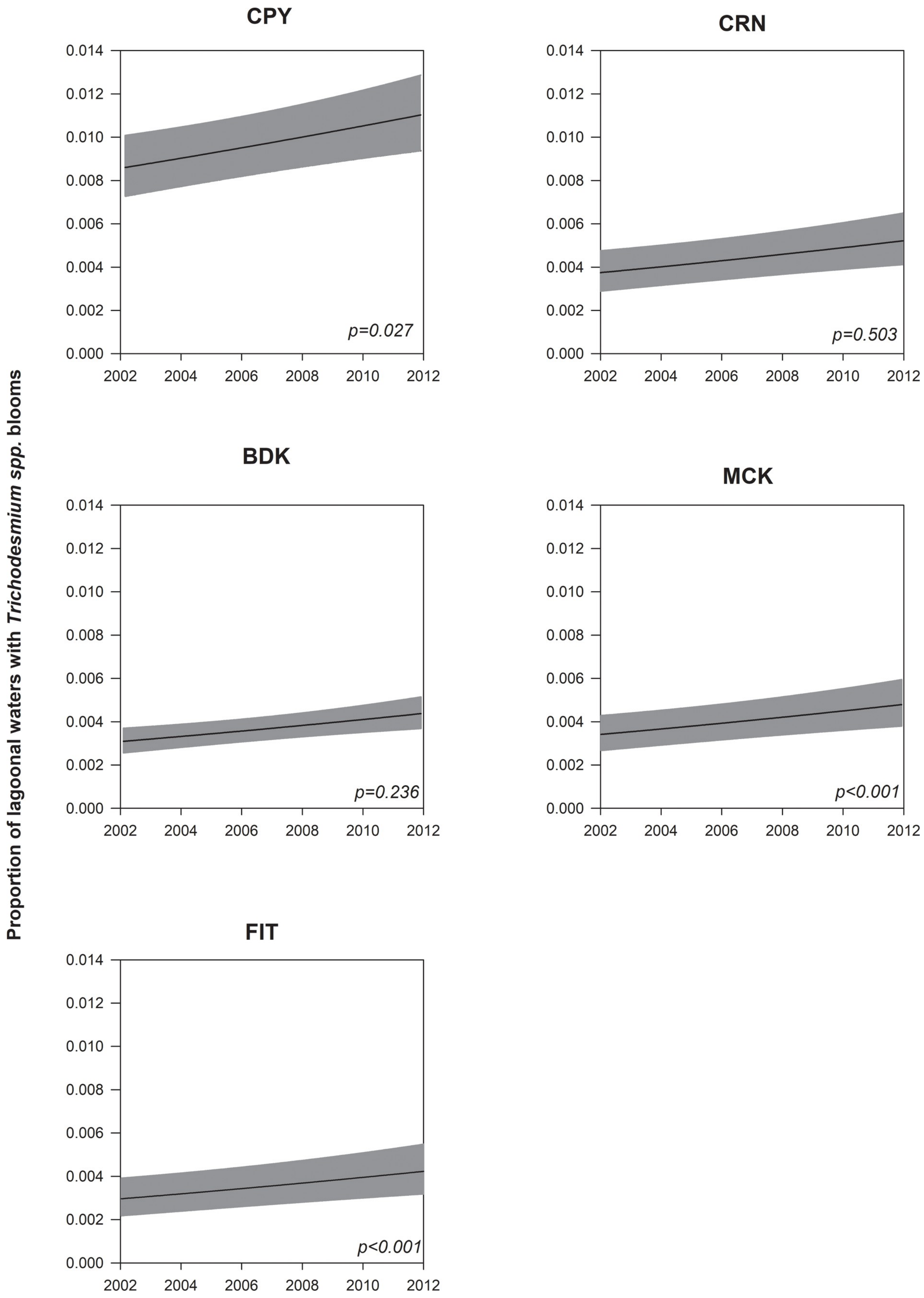
CPY
CRN
BDK
MCK
FIT
Proportion of lagoonal waters with *Trichodesmium spp.* blooms
p=0.027
p=0.503
p=0.236
p<0.001
p<0.001
0.000
0.002
0.004
0.006
0.008
0.010
0.012
0.014
2002
2004
2006
2008
2010
2012

Fig 6. Beta Generalized Additive Model (GAM) outputs modeling the decadal trends in Trichodesmium blooms in each subregion. The y-axis is the proportion of lagoonal waters affected by bloom events in each subregion over the course of the 10-year period. The units are bloom pixels per total (cloud-free) pixels. Confidence intervals for each model are shown as gray bands, and the statistical significance of the trends (p-value) is shown.

https://doi.org/10.1371/journal.pone.0208010.g006

northeastern coast of Australia [78], which might have supported the *Trichodesmium* blooms observed in the northern and central GBR regions of CPY, CRN and BDK in those years. In the GBR, nitrogen (N) fixation by *Trichodesmium* and N inputs from river runoff are proposed as major sources of new N to the system [23, 79], but no study to date has measured the detailed temporal and spatial variability characterizing N fixation activity. From the GAM results, an increase in bloom frequency of *Trichodesmium* occurred over the study's 10-year period (2002–2012), suggesting that their importance as a source of N has also increased over the same period.

In other systems, the extent and densities of *Trichodesmium* aggregates are controlled by nutrient availability, such as phosphorus [4]. Dissolved organic phosphorus (DOP) is the primary form of phosphorus in the GBR [80], and previous studies show that *Trichodesmium* can grow in enriched DOP environments, such as those found in the southern GBR (S1 Fig.) [81]. The concentrations of both Nitrogen and available Phosphorus are generally very low in the GBR (S1 Fig), with a median inorganic Nitrogen:Phosphorus ratio of < 2, which is considerably less than the canonical Redfield Nitrogen:Phosphorus ratio (16:1) measured in phytoplankton biomass [82]. This result suggests that the GBR is severely nitrogen limited and that the nitrogen-fixing *Trichodesmium* has a competitive advantage when compared to that of non-nitrogen-fixing phytoplankton.

The relative quantity of nutrients exported into the lagoon differs significantly between catchments [38] and therefore subregions (S1 Fig.), but the north-south difference in nutrient concentrations was far less pronounced than cross-shelf differences within a subregion [25]. The effect of nutrient fluxes on phytoplankton populations was evident in some of the regions, such as MCK [83], and whereas comparatively little nutrient export occurs during the dry season months, other physical forcing, such as wind and tidal mixing, can play a major role in the cycling of nutrients. The dry tropics catchments export greater quantities of dissolved nitrogen than do the wet tropics catchments, but in both systems, the concentrations are only sufficient for a few days of phytoplankton growth [84].

Dust storms are another important source of nutrient enrichment (Nitrogen, Phosphorus and iron in particular), shifting phytoplankton species composition from pico-cyanobacteria (*Synechococcus*, *Prochlorococcus*) [85, 86] to larger-sized groups such as diatoms and *Trichodesmium*, which may have important implications for other levels of the trophic system. Several studies in other parts of the world highlight the controlling factor of dust storms over periodic *Trichodesmium* blooms and their correlation to colony abundance, acting as iron fertilization that stimulates blooms [87, 88]. Regular dust storms occur over the Australian east coast, and the most notable occurred on October 23–24, 2002, [86] and September 22–24,

Table 4. Average and maximum wind speed (in $m.s^{-1}$) with corresponding surface aggregation sizes (same day; based on daily data).

MCI_{PI}	Average wind speed	Maximum wind speed	Sample size
**1–100**	8.40	16.43	476
**100–500**	7.53	13.00	94
**500–1,000**	6.33	9.00	12
**1,000–4,000**	7.77	12.00	13

https://doi.org/10.1371/journal.pone.0208010.t004

Table 5. Average and maximum wind speed (in m.s^{-1}) two days before MERIS scenes of surface aggregation (based on daily data).

MCI_{PI}	Average wind speed (MERIS-2 days)	Maximum wind speed (MERIS-2 days)	Sample size
1–100	8.38	15.00	476
100–500	7.97	13.00	94
500–1,000	7.75	11.00	12
1,000–4,000	7.93	10.00	13

https://doi.org/10.1371/journal.pone.0208010.t005

2009, as large dust storms (500 km-wide dust plumes) that spread over 2,400 km and 3,500 km (covering the distance Sydney-Cape York), respectively. These storms resulted in phytoplankton blooms, including *Trichodesmium*, mostly in the southern GBR within a week of these events. Such occurrences are typical of the GBR, as it is an ocean region in which a rapid biological response to dust inputs is most likely to be observed [14, 89]. To date, the knowledge about iron cycling in the GBR remains limited, and therefore, to ascertain its role in triggering *Trichodesmium* blooms is currently not possible.

Trichodesmium often forms blooms parallel to the direction of the wind and as drifters, tend to accumulate in convergence zones (e.g., [56]). Low wind speed is strongly associated with *Trichodesmium* aggregations because of the resulting water column stability [14]. In this study, we found an inverse relationship between wind speed and the size of the aggregations, leading us to hypothesize that a wind speed <6 m.s^{-1} is required to observe large ($>2,000$ MCI_{PI}) aggregations.

The GBR is the only large tide-dominated reef in the world, and the tidal energy exerts a strong influence on parts of the shelf and across the coral reef matrix [90], in particular near Broad Sound (MCK; Fig 1) and CPY in which large tidal ranges (up to 9 m) can occur [91]. Shallower water depths (25–50 m) are found in the northern GBR (from 18˚ S northward), whereas the southern GBR is typically much deeper and can reach up to 80 m average depth in some areas, exceeding 100 m near the Capricorn Channel. Thus, in CPY, the increase in water column mixing could hamper the development of regular *Trichodesmium* blooms. In other regions, however, such as CRN and BDK, tidal flows do not significantly contribute to water transport [92]. Because wind-driven resuspension occurs mostly between May and October [93], a frontal separation may be created between the lagoon and the coastal zone, thereby trapping terrestrial material and nutrients in nearshore waters and favoring the appearance of *Trichodesmium* blooms. During wet season flood events, large volumes of freshwater runoff entering the GBR reduce light penetration and decrease salinity levels (along the coast in particular, $<<33$ ppt; [94, 95]), and despite being a euryhaline genus, maximum growth for *Trichodesmium* tends to occur at salinities of 33–37 ppt. Thus, we hypothesize that the tropical wet season conditions are suboptimal for *Trichodesmium* growth in the GBR, in particular near large catchments such as FIT and BDK (Fig 1).

Several interacting environmental factors are likely involved in affecting the probability of the occurrence and extent of *Trichodesmium* blooms (e.g., surface mixing by wind or/and tides, nutrient inputs from river plumes or dust storms, and grazer density, among others) beyond the spatial, temporal, and thermal effects assessed in this study. However, despite the complex dynamics of *Trichodesmium* blooms, the combined effects of spatial, temporal, and SST factors alone may account for 14% of the probability of a bloom occurring and 18.5% of the bloom extent when a bloom occurs.

MCI_{PI} limitations

MERIS MCI_{PI} was an appropriate algorithm for the detection and monitoring of surface expressions in the study region. Compared with this study, the match-up analysis by [56] had

higher detection success at 85% but used a much finer resolution of 250-m pixel size from MODIS-Aqua and therefore was more likely to detect small and filamentous patches. This MODIS-based analysis is in contrast to the present study's validation for which the MERIS RR 1.2-km pixel size was used (MERIS FR imagery was irregularly acquired over Australia and therefore was not useable as a consistent time-series). The MODIS algorithm by [56] also relied on an atmospheric correction option only available in an outdated SeaDAS version; therefore, it was not possible to extend that analysis on the entire MODIS time-series. The limited information, other than the location and datum, provided by the field observation dataset also likely affected the quality of the retrieval analysis. The validation exercise in the present study was used for a general assessment of this algorithm. We recommend that additional criteria such as the size of the aggregations and a confirmation of the genus (*i.e.*, is it *Trichodesmium* sp.?) should be considered to complement a future *Trichodesmium*-specific field database.

The MCI has been used in several previous studies for the detection, monitoring and analysis of the seasonal dynamics, spatial distribution, and coverage of bloom surface expressions in a large range of freshwater (e.g., [96, 97]) and marine (e.g., [55]) environments. This index is versatile and with low penetration depth (<1 m) is fairly insensitive to bottom reflectance (e.g., [98]), which is a significant advantage in this relatively shallow coastal system in which Secchi depths can reach the seafloor of the GBR lagoon [32]. The MCI also has limitations. The index has a limited application for low (Chl-a<10 $\mu g.L^{-1}$) phytoplankton biomass conditions, as observed in the GBR [99]. In this study, the MCI was not employed to detect phytoplankton blooms based on their Chl-a concentration but on their surface expressions for which the MCI is suitable. Our previous study in the Cairns subregion combined the use of the FLH, Chl-a and MCI MERIS products, which showed that MCI and Chl-a were mostly uncorrelated, thereby confirming that MCI was predominantly responding to surface bloom expressions in the GBR [31]. Another recently documented limitation is the sensitivity of the MCI to inorganic particles (suspended sediment) [100], but we are confident this factor did not affect our results because the surface bloom observations occurred well outside the runoff (wet) season, when large sediment plumes may be observed. The use of the MERIS MCI_{PI} alone, however, is not sufficient to confirm the genus of the phytoplankton bloom. For example, in the southern GBR (FIT), few instances of the surface expressions identified in this work as *Trichodesmium* sp. surface expressions could in fact be *Lyngbya majuscula*, a benthic cyanobacterium, or *Hincksia sordida*, a filamentous brown algae, as both are known to form surface aggregations in Southeast Queensland coastal waters (MCK, FIT) [101, 102]. Hence, as no other taxa are likely to lead to consistent and significant blooms in the GBR that would trigger MCI_{PI}, we can confidently interpret the MCI_{PI} patterns as *Trichodesmium* sp. surface expressions.

Finally, an inherent bias exists in ocean color satellite observations due to cloud cover that hampers the collection of observations, in particular during the wet season. This variability must be acknowledged when analyzing the frequency of observed clear sky, pixel-based blooms in tropical and subtropical regions. Although this study was primarily based on monthly composites, *i.e.*, the aggregation of several scenes over the course of each month, the analysis of time-series composing >1,000 scenes for each subregion should compensate for this bias.

Concluding remarks

This study used MERIS MCI_{PI} to assess the spatio-temporal dynamics of *Trichodesmium* surface expressions over the entire GBR lagoonal waters during the decade-long MERIS mission.

To date, this study is the only one that evaluated both the spatial and seasonal distributions of *Trichodesmium* blooms over the entire GBR and for a decade-long period.

The results showed a north to south gradient in bloom sizes (increasing from CPY to FIT), in bloom timings, appearing later in the year, from CPY in July to FIT in October-November (Table 4), and in bloom frequencies, with the largest and most frequent surface blooms occurring in FIT, the southernmost subregion. A temperature of 24˚C and a wind speed <6 $m.s^{-1}$ were associated with larger events. Nitrogen fixation by *Trichodesmium* remains an incompletely constrained component of the GBR nitrogen budget, particularly in the subsurface [20], and our results suggest an increased importance of *Trichodesmium* as a source of new nitrogen to the GBR.

Recent modeling efforts are attempting to predict the distribution and growth of *Trichodesmium* from physiological- and remote sensing-based models incorporated into larger hydrodynamic and biogeochemical models of the Great Barrier Reef lagoon through eReefs (https://ereefs.org.au/ereefs) to inform environmental management of this region [103, 104]. By analyzing the phenology of *Trichodesmium* surface bloom aggregations along the GBR over a decade from satellite imagery, the findings of our work directly benefit this ecosystem modeling effort. The recent launch of multispectral Sentinel-2 MSI (launched in June 2015) and Sentinel-3 OLCI (launched in February 2016) satellite sensors that have matching wavebands to those of the MERIS sensor will allow this research to continue.

Supporting information

S1 Fig. Summary of the biological, chemical and physical properties of surface water samples for the five subregions. Based on averaged values from the period 2002 to 2013 for temperature, chlorophyll-a (Chl-a), particulate organic carbon (POC), nitrogen (PN), and phosphate (PP), dissolved inorganic nitrogen (DIN) and organic phosphorus (DOP), and total dissolved nitrogen (TDN) and phosphorus (TDP).
(TIF)

S2 Fig. Time-series of monthly averaged valid satellite observations for the five subregions (i.e., not masked by quality flags or other criteria). Subregions are ordered from north to south, and the y-axis scale is uniform for all plots.
(TIF)

S3 Fig. Time-series of monthly median MCI background value, b, for the five subregions (subregions are ordered from north to south). Vertical dashed lines represent 6-month periods for all plots.
(TIF)

S4 Fig. Monthly climatology of surface bloom occurrences (%MCIPI) for CPY over the period April 2002-April 2012.
(TIF)

S5 Fig. Monthly climatology of surface bloom occurrences (%MCIPI) for CRN over the period April 2002-April 2012.
(TIF)

S6 Fig. Monthly climatology of surface bloom occurrences (%MCIPI) for BDK over the period April 2002-April 2012.
(TIF)

S7 Fig. Monthly climatology of surface bloom occurrences (%MCIPI) for MCK over the period April 2002-April 2012.
(TIF)

S1 Table. Bernoulli GAM evaluating the effect of SST, region, time of year (Julian date) by region, and interannual variability (year) on the likelihood of a Trichodesmium bloom occurring. Significant p-values ($\alpha = 0.05$) are indicated in bold.
(DOCX)

S2 Table. Beta GAM evaluating the effect of SST, region, time of year (Julian date) by region, and interannual variability (year) on the extent Trichodesmium blooms when they occur. Significant p-values ($\alpha = 0.05$) are indicated in bold.
(DOCX)

S1 Dataset. MCI_GreatBarrierReef_20022012.csv.
(CSV)

Acknowledgments

For this study, DBP was partly supported by a NAMRA post-doctoral fellowship and by CSIRO Oceans and Atmosphere funding. CNR, the RITMARE Flagship Project and the European Union (FP7-427 People Co-funding of Regional, National and International Programmes, GA n. 600407) supported VEB. Collection of the nutrient data presented in this study was obtained with support from the Great Barrier Reef Marine Park Authority (GBRMPA), through funding from the Australian Government's Caring for our Country Program and from the Australian Institute of Marine Science. The authors acknowledge the data providers: The European Space Agency (ESA) and ACRI-ST (www.acri-st.fr/) for providing the MERIS RR dataset; The Bureau of Meteorology (BoM; www.bom.gov.au), IMOS (Integrated Marine Observing System; http://imos.org.au) and eReefs (http://ereefs.org.au). The authors are grateful to: Elsevier Language Editing Services for providing editing support; Jonathan Hodge (CSIRO Oceans and Atmosphere) for providing guidance in extracting the wind data from eReefs; CSIRO Data61 (Ecosciences Precinct) for advice for parts of the data analysis, and Dr Phillip Ford, Dr Peter Rothlisberg and Dr Tim Malthus (CSIRO Oceans and Atmosphere) for comments on previous versions of the manuscript. All the processing of the ESA MERIS imagery was performed with the Sentinel Application Platform (SNAP) (Brockmann Consult).

Author Contributions

Conceptualization: David Blondeau-Patissier, Vittorio Ernesto Brando, Susannah M. Leahy.

Data curation: David Blondeau-Patissier.

Formal analysis: David Blondeau-Patissier, Susannah M. Leahy.

Investigation: David Blondeau-Patissier.

Methodology: David Blondeau-Patissier, Vittorio Ernesto Brando, Susannah M. Leahy, Arnold G. Dekker.

Resources: Christian Lønborg.

Software: David Blondeau-Patissier, Susannah M. Leahy.

Supervision: Vittorio Ernesto Brando, Arnold G. Dekker.

Visualization: David Blondeau-Patissier.

Writing – original draft: David Blondeau-Patissier.

Writing – review & editing: David Blondeau-Patissier, Vittorio Ernesto Brando, Christian Lønborg, Susannah M. Leahy, Arnold G. Dekker.

References

1. Berman-Frank I, Lundgren P, Chen Y-B, Kupper H, Kolber Z, Bergman B, et al. Segregation of Nitrogen Fixation and Oxygenic Photosynthesis in the Marine Cyanobacterium Trichodesmium. Science. 2001; 294:1534–7. https://doi.org/10.1126/science.1064082 PMID: 11711677
2. Westberry TK, Siegel DA. Spatial and temporal distribution of Trichodesmium blooms in the world's oceans. Global Biogeochemical Cycles. 2006; 20(4). https://doi.org/10.1029/2005GB002673
3. Capone DG, Zehr JP, Paerl HW, Bergman B, Carpenter EJ. Trichodesmium, a globally significant marine cyanobacterium. Science. 1997; 276(5316):1221–9.
4. Sohm JA, Webb EA, Capone DG. Emerging patterns of marine nitrogen fixation. Nature. 2011; 9 (7):499–508.
5. Spatharis S, Skliris N, Meziti A, Kormas Konstantinos A. First record of a *Trichodesmium erythraeum* bloom in the Mediterranean Sea. Canadian Journal of Fisheries and Aquatic Sciences. 2012; 69 (8):1444–55. https://doi.org/10.1139/f2012-020
6. Rees AP, Tait K, Widdicombe CE, Quartly GD, McEvoy AJ, Al-Moosawi L. Metabolically active, non-nitrogen fixing, Trichodesmium in UK coastal waters during winter. Journal of plankton research. 2016; 38(3):673–8. https://doi.org/10.1093/plankt/fbv123 PMID: 27274100
7. Bell PRF, Uwins PJR, Elmetri I, Phillips JA, Fu F-X, Yago AJE. Laboratory culture studies of Trichodesmium isolated from the Great Barrier Reef Lagoon, Australia. Hydrobiologia. 2005; 532(1):9–21. https://doi.org/10.1007/s10750-004-8768-1
8. Orcutt KM, Gundersen K, Rasmussen U. Another look at green Trichodesmium colonies. Limnology and Oceanography. 2008; 53:2049–51.
9. Clevers JGPW, de Jong SM, Epema GF, van der Meer F, Bakker WH, Skidmore AK, et al. MERIS and the red-edge position. International Journal of Applied Earth Observation and Geoinformation. 2001; 3 (4):313–20. https://doi.org/10.1016/s0303-2434(01)85038-8
10. McKinna LIW. Three decades of ocean-color remote-sensing Trichodesmium spp. in the World's oceans: A review. Progress in Oceanography. 2014; 131:177–99. https://doi.org/10.1016/j.pocean.2014.12.013
11. Dupouy C, Petit M, Dandonneau Y. Satellite detected cyanobacteria bloom in the southwestern tropical Pacific. Implication for nitrogen fixation. International Journal of Remote Sensing. 1988; 8(3):389–96.
12. Subramaniam A, Carpenter EJ. An empirically derived protocol for the detection of blooms of the marine cyanobacteria *Trichodesmium* using CZCS imagery. International Journal of Remote Sensing. 1994; 15(8):1559–69. https://doi.org/10.1080/01431169408954191
13. Mulholland MR. The fate of nitrogen fixed by diazotrophs in the ocean. Biogeosciences. 2007; 4:37–51. https://doi.org/10.5194/bg-4-37-2007
14. Furnas M. Pelagic Trichodesmium (= Oscillatoria) in the Great Barrier Reef region. In: Carpenter EJ, Capone DG, Reuter JG, editors. Marine Pelagic Cyanobacteria: Trichodesmium and other Diazotrophs. NATO ASI Series. 362. Netherlands: Springer; 1992. p. 265–72.
15. Wilson C, Qiu X. Global distribution of summer chlorophyll blooms in the oligotrophic gyres. Progress in Oceanography. 2008; 78(2):107–34. https://doi.org/10.1016/j.pocean.2008.05.002
16. Hutchins DA, Fu FX, Zhang Y, Warner ME, Feng Y, Portune K, et al. CO2 control of Trichodesmium N2 fixation, photosynthesis, growth rates, and elemental ratios: Implications for past, present, and future ocean biogeochemistry. Limnology and Oceanography. 2007; 52(4):1293–304. https://doi.org/10.4319/lo.2007.52.4.1293
17. Bergman B, Sandh G, Lin S, Larsson J, Carpenter EJ. Trichodesmium–a widespread marine cyanobacterium with unusual nitrogen fixation properties. FEMS Microbiology Reviews. 2013; 37(3):286–302. https://doi.org/10.1111/j.1574-6976.2012.00352.x PMID: 22928644
18. Villareal TA, Adornato L, Wilson C, Schoenbaechler CA. Summer blooms of diatom-diazotroph assemblages and surface chlorophyll in the North Pacific gyre: A disconnect. Journal of Geophysical Research. 2011; 116(C3):C03001. https://doi.org/10.1029/2010jc006268

19. Karl DM, Letelier R, Hebel D, Bird D, Winn C. Trichodesmium blooms and new nitrogen in the North Pacific gyre. Marine pelagic cyanobacteria: Trichodesmium and other diazotrophs1992. p. 219–37.
20. Olson EM, McGillicuddy DJ, Dyhrman ST, Waterbury JB, Davis CS, Solow AR. The depth-distribution of nitrogen fixation by Trichodesmium spp. colonies in the tropical–subtropical North Atlantic. Deep Sea Research Part I: Oceanographic Research Papers. 2015; 104:72–91. https://doi.org/10.1016/j.dsr.2015.06.012
21. Sheridan CC, Steinberg DK, Kling GW. The microbial and metazoan community associated with colonies of Trichodesmium spp.: a quantitative survey. Journal of Plankton Research. 2002; 24(9):913–22. https://doi.org/10.1093/plankt/24.9.913
22. Brando VE, Schroeder T, King E, Dyce P. Reef Rescue Marine Monitoring Program: Using remote sensing for GBR-wide water quality—CSIRO Report to the Great Barrier Reef Marine Park Authority. CSIRO, 2015.
23. Furnas M, Alongi D, McKinnon D, Trott L, Skuza M. Regional-scale nitrogen and phosphorus budgets for the northern (14˚S) and central (17˚S) Great Barrier Reef shelf ecosystem. Continental Shelf Research. 2011; 31(19–20):1967–90. http://dx.doi.org/10.1016/j.csr.2011.09.007.
24. Marshall SM. The production of microplankton in the Great Barrier Reef. Scientific Report. London, England: British Museum (Natural History), 1933 Contract No.: 5.
25. Revelante N, Williams WT, Bunt JS. Temporal and spatial distribution of diatoms, dinoflagellates and Trichodesmium in waters of the Great Barrier Reef. Journal of Experimental Marine Biology and Ecology. 1982; 63:27–45. https://doi.org/10.1016/0022-0981(82)90048-X
26. Dupouy C, Frouin R, Tedetti M, Maillard M, Rodier M, Lombard F, et al. Diazotrophic Trichodesmium influence on ocean color and pigment composition in the South West tropical Pacific. Biogeosciences Discuss. 2018:1–43. https://doi.org/10.5194/bg-2017-570
27. Rousset G, De Boissieu F, Menkes CE, Lefèvre J, Frouin R, Rodier M, et al. Remote Sensing of Trichodesmium spp. mats in the Western Tropical South Pacific. Biogeosciences Discuss. 2018; 2018:1–29. https://doi.org/10.5194/bg-2017-571
28. White AE, Watkins-Brandt KS, Church MJ. Temporal Variability of Trichodesmium spp. and Diatom-Diazotroph Assemblages in the North Pacific Subtropical Gyre. Frontiers in Marine Science. 2018; 5 (27). https://doi.org/10.3389/fmars.2018.00027
29. Blondeau-Patissier D, Schroeder T, Brando VE, Maier SW, Dekker AG, Phinn S. ESA-MERIS 10-Year Mission Reveals Contrasting Phytoplankton Bloom Dynamics in Two Tropical Regions of Northern Australia. Remote Sens. 2014; 6(4):2963–88. https://doi.org/10.3390/rs6042963
30. Gower J, King S, Borstad G, Brown L. Detection of intense plankton blooms using the 709 nm band of the MERIS imaging spectrometer. INTERNATIONAL JOURNAL OF REMOTE SENSING. 2005; 26 (9):2005–12. https://doi.org/10.1080/01431160500075857
31. Furnas M. Catchments and Corals: Terrestrial runoff to the Great Barrier Reef. Townsville, Aus.: CRC/AIMS; 2003. 335- p.
32. Weeks S, Werdell PJ, Schaffelke B, Canto M, Lee Z, Wilding JG, et al. Satellite-Derived Photic Depth on the Great Barrier Reef: Spatio-Temporal Patterns of Water Clarity. Remote Sensing. 2012; 4 (12):3781–95. Epub Detecting. https://doi.org/10.3390/rs4123781
33. Schroeder T, Devlin MJ, Brando VE, Dekker AG, Brodie JE, Clementson LA, et al. Inter-annual variability of wet season freshwater plume extent into the Great Barrier Reef lagoon based on satellite coastal ocean colour observations. Marine Pollution Bulletin. 2012; 65(4–9):210–23. https://doi.org/10.1016/j.marpolbul.2012.02.022 PMID: 22459495
34. Lough J. Climate and climate change on the Great Barrier Reef. In: Johnson JE, Marshall PA, editors. Climate Change and Great Barrier Reef. Townsville (Aus.): Great Barrier Reef Marine Park Authority and Australian Greenhouse Office; 2007. p. 15–50.
35. Mackenzie R, Robinson J, Dent J, Scheltinga D. Sustainable Land Management and Wetlands Conservation on Freehold and Leasehold Land in the Great Barrier Reef Catchment. Brisbane: the Department of the Environment, Water, Heritage and the Arts, 2005.
36. Prange J, Johnson JE, Morris S. Reef Water Quality Protection Plan Marine Monitoring Program 2007/2008 Summary Report. Cairns: Reef and Rainforest Research Centre Limited, 2009.
37. Devlin MJ, Brodie J. Terrestrial discharge into the Great Barrier Reef Lagoon: nutrient behavior in coastal waters. Marine Pollution Bulletin. 2005; 51(1–4):9–22. https://doi.org/10.1016/j.marpolbul.2004.10.037 PMID: 15757704
38. Robson BJ, Dourdet V. Prediction of sediment, particulate nutrient and dissolved nutrient concentrations in a dry tropical river to provide input to a mechanistic coastal water quality model. Environmental Modelling and Software. 2015; 63(0):97–108. http://dx.doi.org/10.1016/j.envsoft.2014.08.009.

39. Webster IT, Ford PW. Delivery, deposition and redistribution of fine sediments within macrotidal Fitzroy Estuary/Keppel Bay: Southern Great Barrier Reef, Australia. Continental Shelf Research. 2010; 30 (7):793–805. https://doi.org/10.1016/j.csr.2010.01.017
40. Schaffelke B, Carleton J, Skuza M, Zagorskis I, Furnas MJ. Water quality in the inshore Great Barrier Reef lagoon: Implications for long-term monitoring and management. Marine Pollution Bulletin. 2012; 65(4–9):249–60. https://doi.org/10.1016/j.marpolbul.2011.10.031 PMID: 22142496
41. Lønborg C, Doyle J, Furnas M, Menendez P, Benthuysen JA, Carreira C. Seasonal organic matter dynamics in the Great Barrier Reef lagoon: Contribution of carbohydrates and proteins. Continental Shelf Research. 2017; 138:95–105. https://doi.org/10.1016/j.csr.2017.01.010.
42. Gower J, King S, Goncalves P. Global monitoring of plankton blooms using MERIS MCI. International Journal of Remote Sensing. 2008; 29(21):6209–16. https://doi.org/10.1080/01431160802178110
43. Blondeau-Patissier D, Brando VE, Oubelkheir K, Dekker AG, Clementson LA, Daniel P. Bio-optical variability of the absorption and scattering properties of the Queensland inshore and reef waters, Australia. Journal of Geophysical Research. 2009; 114(C05003):2156–202. https://doi.org/10.1029/2008JC005039
44. Brodie J, De'ath G, Devlin M, Furnas M, Wright M. Spatial and temporal patterns of near-surface chlorophyll a in the Great Barrier Reef lagoon. Marine and Freshwater Research. 2007; 58(4):342–53. https://doi.org/10.1071/MF06236
45. Gower J, King SA. Distribution of floating Sargassum in the Gulf of Mexico and the Atlantic Ocean mapped using MERIS. International Journal of Remote Sensing. 2011; 32(7):1917–29. https://doi.org/10.1080/01431161003639660
46. Gower J, Hu C, Borstad G, King S. Ocean Color Satellites show extensive lines of floating Sargassum in the Gulf of Mexico. IEEE Transaction on Geoscience and Remote Sensing. 2006; 44:3619–25. https://doi.org/10.1109/TGRS.2006.882258
47. Gower J, Young E, King S. Satellite images suggest a new Sargassum source region in 2011. Remote Sensing Letters. 2013; 4(8):764–73. https://doi.org/10.1080/2150704X.2013.796433
48. Gower J, King S, Young E. Global remote sensing of Trichodesmium. International Journal of Remote Sensing. 2014; 35(14):5459–66. https://doi.org/10.1080/01431161.2014.926422
49. Blondeau-Patissier D, Gower JFR, Dekker AG, Phinn SR, Brando VE. A review of ocean color remote sensing methods and statistical techniques for the detection, mapping and analysis of phytoplankton blooms in coastal and open oceans. Progress in Oceanography. 2013; 123:123–44. http://dx.doi.org/10.1016/j.pocean.2013.12.008.
50. Moradi M. Comparison of the efficacy of MODIS and MERIS data for detecting cyanobacterial blooms in the southern Caspian Sea. Marine Pollution Bulletin. 2014;(0). http://dx.doi.org/10.1016/j.marpolbul.2014.06.053.
51. Binding CE, Greenberg TA, Bukata RP, Smith DE, Twiss MR. The MERIS MCI and its potential for satellite detection of winter diatom blooms on partially ice-covered Lake Erie. Journal of Plankton Research. 2012; 34(6):569–73. https://doi.org/10.1093/plankt/fbs021
52. Fomferra N, Brockmann C, editors. BEAM—The ENVISAT MERIS and AATSR Toolbox. The MERIS-(A)ATSR workshop; 2005 26–30 September; Frascati (It.): ESA.
53. Binding CE, Greenberg TA, Bukata RP. The MERIS Maximum Chlorophyll Index; its merits and limitations for inland water algal bloom monitoring. Journal of Great Lakes Research. 2013; 39(Sup. 1):100–7. http://dx.doi.org/10.1016/j.jglr.2013.04.005.
54. Gower J, King S, editors. On the importance of a band at 709 nm. 2nd MERIS-AATSR Workshop; 2008 Sept 23 2008; Frascati (It.): ESA.
55. Gower J, King S. A Global Survey of Intense Surface Plankton Blooms and Floating Vegetation using MERIS MCI. In: Tang D, editor. Remote Sensing of the Changing Oceans. 1st ed: Springer; 2011. p. 99–121.
56. McKinna LIW, Furnas MJ, Ridd PV. A simple, binary classification algorithm for the detection of Trichodesmium spp. within the Great Barrier Reef using MODIS imagery. Limnology and Oceanography-Methods. 2011; 9:50–66. https://doi.org/10.4319/lom.2011.9.50
57. Jassby AD, Cloern JE. Package 'wq'—Some tools for exploring water quality monitoring data. In: R-CRAN, editor. 0.3–4 ed: R-CRAN; 2011.
58. Zuur AF, Saveliev AA, Leno EN. Zero Inflated Models and Generalized Linear Mixed Models with R: Highland Statistics Limited; 2012.
59. Brodie JE, Waterhouse J, Schaffelke B, Kroon FJ, Thorburn P, Rolfe J, et al. 2013 Scientific Consensus Statement: Land use impacts on Great Barrier Reef water quality and ecosystem condition. 2013.

60. Leahy SM, Kingsford MJ, Steinberg CR. Do clouds save the great barrier reef? satellite imagery elucidates the cloud-SST relationship at the local scale. PLoS ONE. 2013; 8(7):e70400. https://doi.org/10.1371/journal.pone.0070400 PMID: 23894649

61. Ferrari S, Cribari-Neto F. Beta Regression for Modelling Rates and Proportions. Journal of Applied Statistics. 2004; 31(7):799–815. https://doi.org/10.1080/0266476042000214501

62. Luo Y-W, Lima ID, Karl DM, Deutsch CA, Doney SC. Data-based assessment of environmental controls on global marine nitrogen fixation. Biogeosciences. 2014; 11:691–708. https://doi.org/10.5194/bg-11-691-2014

63. Snow JT, Schlosser C, Woodward EMS, Mills MM, Achterberg EP, Mahaffey CA, et al. Environmental controls on the biogeography of diazotrophy and Trichodesmium in the Atlantic Ocean. Global Biogeochemical Cycles. 2015; 29. https://doi.org/10.1002/2015GB005090

64. Fu F-X, Bell PRF. Effect of salinity on growth, pigmentation, N2 fixation and alkaline phosphatase activity of cultured Trichodesmium sp. Marine Ecology Progress Series 2003; 257:69–76.

65. Bell PRF, Fu F-X. Effect of light on growth, pigmentation and N sub(2) fixation of cultured Trichodesmium sp. from the Great Barrier Reef lagoon. Hydrobiologia. 2005; 543(1):25–35.

66. Breitbarth E, Wohlers J, Kläs J, La Roche J, Peeken I. Nitrogen fixation and growth rates of Trichodesmium IMS-101 as a function of light intensity. Marine Ecology-Progress Series. 2008; 359:25–36. Epub May 5. https://doi.org/10.3354/meps07241

67. Mills MM, Ridame C, Davey M, La Roche J, Geider RJ. Iron and phosphorus co-limit nitrogen fixation in the eastern tropical North Atlantic. Nature. 2004; 429:292–4. https://doi.org/10.1038/nature02550 PMID: 15152251

68. O'Neil JM, Roman MR. Ingestion of the cyanobacterium Trichodesmium spp. by pelagic harpacticoid copepods Macrosetella, Miracia and Oculosetella. In: Ferrari F, Bradley B, editors. Ecology and Morphology of Copepods. Developments in Hydrobiology. 102: Springer Netherlands; 1994. p. 235–40.

69. Hewson I, Govil SR, Capone DG, Carpenter EJ, Fuhrman JA. Evidence of Trichodesmium viral lysis and potential significance for biogeochemical cycling in the oligotrophic ocean. Aquatic Microbial Ecology. 2004; 36(1):1–8. https://doi.org/10.3354/ame036001

70. Rodier M, Le Borgne R. Population dynamics and environmental conditions affecting Trichodesmium spp. (filamentous cyanobacteria) blooms in the south-west lagoon of New Caledonia. Journal of Experimental Marine Biology and Ecology. 2008; 358(1):20–32. https://doi.org/10.1016/j.jembe.2008.01.016

71. Robarts RD, Zohary T. Temperature effects on photosynthetic capacity, respiration and growth rates of bloom-forming cyanobacteria. New Zeland Journal of Marine and Freshwater Research. 1987; 21:391–9.

72. Fu F-X, Yu E, Garcia NS, Gale J, Luo Y, Webb EA, et al. Differing responses of marine N2 fixers to warming and consequences for future diazotroph community structure. Aquatic Microbial Ecology. 2014; 72:33–46. https://doi.org/10.3354/ame01683

73. Srokosz MA, Quartly GD. The Madagascar Bloom—a serendipitous study. Journal of Geophysical Research. 2013; 118(1):14–25. https://doi.org/10.1029/2012JC008339

74. Gower J, King S. Satellite Water Colour Observations in African Seas. In: Barale V, Grade M, editors. Remote Sensing of the African Seas. Netherlands: Springer; 2014. p. 31–53.

75. Ward BA, Dutkiewicz S, Moore CM, Follows MJ. Iron, phosphorus, and nitrogen supply ratios define the biogeography of nitrogen fixation. Limnology and Oceanography. 2013; 58(6):2059–75. https://doi.org/10.4319/lo.2013.58.6.2059c

76. Rijkenberg MJA, Langlois RJ, Mills MM, Patey MD, Hill PG, Nielsdottir MC, et al. Environmental Forcing of Nitrogen Fixation in the Eastern Tropical and Sub-Tropical North Atlantic Ocean. PLoS One. 2011; 6(12):e28989. https://doi.org/10.1371/journal.pone.0028989 PMID: 22174940

77. Tyrrell T, Maranon E, Poulton AJ, Bowie AR, Harbour DS, Woodward EMS. Large-scale latitudinal distribution of Trichodesmium spp. in the Atlantic Ocean. Journal of Plankton Research. 2003; 25 (4):405–16. https://doi.org/10.1093/plankt/25.4.405

78. Thompson PA, Bonham P, Thomson P, Rochester W, Doblin MA, Waite AM, et al. Climate variability drives plankton community composition changes: the 2010–2011 El Niño to La Niña transition around Australia. Journal of Plankton Research. 2015; 37(5):966–84. https://doi.org/10.1093/plankt/fbv069

79. Messer LF, Brown MV, Furnas MJ, Carney RL, McKinnon AD, Seymour JR. Diversity and Activity of Diazotrophs in Great Barrier Reef Surface Waters. Frontiers in Microbiology. 2017; 8(967). Epub Jun 7. https://doi.org/10.3389/fmicb.2017.00967 PMID: 28638369

80. Lønborg C, Álvarez–Salgado XA, Duggan S, Carreira C. Organic matter bioavailability in tropical coastal waters: The Great Barrier Reef. Limnology and Oceanography. 2017; 63(2):1015–35. https://doi.org/10.1002/lno.10717

81. White AE, Karl DM, jörkman K, Beversdorf LJ, Letelier RM. Production of organic matter by *Trichodesmium* IMS101 as a function of phosphorus source. Limnology and Oceanography. 2010; 55(4):1755–67.
82. Redfield AC, Ketchum BH, Richards FA. The influence of organisms on the composition of seawater. In: Hill MN, editor. 2. New York (USA): Interscience Publishers; 1963. p. 26–77.
83. Brodie JE, Devlin M, Haynes D, Waterhouse J. Assessment of the eutrophication status of the Great Barrier Reef lagoon (Australia). Biogeochemistry. 2011; 106:281–302. https://doi.org/10.1007/s10533-010-9542-2
84. Brodie J, Waterhouse J, Schaffelke B, Kroon FJ, Thorburn P, Rolfe J, et al. 2013 Scientific Consensus Statement: Land use impacts on Great Barrier Reef water quality and ecosystem condition. 2014.
85. Crosbie ND, Furnas MJ. Abundance, distribution and flow-cytometric characterization of picophytoprokaryote populations in central (17˚S) and southern (20˚S) shelf waters of the Great Barrier Reef. Journal of Plankton Research. 2001; 23(8):809–28. https://doi.org/10.1093/plankt/23.8.809
86. Shaw EC, Gabric AJ, McTainsh GH. Impacts of aeolian dust deposition on phytoplankton dynamics in Queensland coastal waters. Marine and Freshwater Research. 2008; 59(11):951–62. https://doi.org/10.1071/MF08087
87. Lenes JM, Darrow BA, Walsh JJ, Prospero JM, He R, Weisberg RH, et al. Saharan dust and phosphatic fidelity: A three-dimensional biogeochemical model of Trichodesmium as a nutrient source for red tides on the West Florida Shelf. Continental Shelf Research. 2008; 28(9):1091–115. https://doi.org/10.1016/j.csr.2008.02.009
88. Orcutt KM, Lipschultz F, Gundersen K, Arimoto R, Michaels AF, Knap AH, et al. A seasonal study of the significance of N2 fixation by Trichodesmium spp. at the Bermuda Atlantic Time-series Study (BATS) site. Deep Sea Research Part II: Topical Studies in Oceanography. 2001; 48(8–9):1583–608.
89. Cropp RA, Gabric AJ, Levasseur M, McTainsh GH, Bowie A, Hassler CS, et al. The likelihood of observing dust-stimulated phytoplankton growth in waters proximal to the Australian continent. Journal of Marine Systems. 2013;117–118:43–52. https://doi.org/10.1016/j.jmarsys.2013.02.013
90. Wolanski E, Spagnol S. Sticky waters in the Great Barrier Reef. Estuarine, Coastal and Shelf Science. 2000; 50(1):27–32. https://doi.org/10.1006/ecss.1999.0528
91. Burrage DM, Steinberg CR, Skirving WJ, Kleypas JA. Mesoscale Circulation Features of the Great Barrier Reef Region Inferred from NOAA Satellite Imagery. Remote Sensing Of Environment. 1996; 56(1):21–41.
92. Andrews JC, Clegg S. Coral Sea circulation and transport deduced from modal information models. Deep Sea Research Part I: Oceanographic Research Papers. 1989; 36(6):957–74. https://doi.org/10.1016/0198-0149(89)90037-X
93. Hutchings P, Kingsford M, Hoegh-Guldberg O. The Great Barrier Reef: Biology, Environment and Management: CSIRO; 2008. 392- p.
94. Revelante N, Gilmartin M. Dynamics of Phytoplankton in the Great Barrier Reef Lagoon. Journal of Plankton Research. 1982; 4(1):47–76.
95. Fabricius KE, Logan M, Weeks S, Brodie J. The effects of river run-off on water clarity across the central Great Barrier Reef. Marine Pollution Bulletin. 2014; 84(1–2):191–200. http://dx.doi.org/10.1016/j.marpolbul.2014.05.012. PMID: 24863415
96. Alikas K, Kangro K, Reinart A. Detecting cyanobacterial blooms in large North European lakes using the Maximum Chlorophyll Index. Oceanologia. 2010; 52(2):237–57.
97. Binding CE, Greenberg TA, Bukata RP. An analysis of MODIS-derived algal and mineral turbidity in Lake Erie. Journal of Great Lakes Research. 2012; 38(1):107–16. http://dx.doi.org/10.1016/j.jglr.2011.12.003.
98. Binding CE, Greenberg TA, Jerome JH, Bukata RP, Letourneau G. An assessment of MERIS algal products during an intense bloom in Lake of the Woods. Journal of Plankton Research. 2010; 33(5):793–806. https://doi.org/10.1093/plankt/fbq133
99. Binding CE, Greenberg TA, Bukata RP. The MERIS Maximum Chlorophyll Index; its merits and limitations for inland water algal bloom monitoring. Journal of Great Lakes Research. 2013; 39(Sup. 1):100–7. http://dx.doi.org/10.1016/j.jglr.2013.04.005.
100. Qi L, Hu C, Duan H, Zhang Y, Ma R. Influence of Particle Composition on Remote Sensing Reflectance and MERIS Maximum Chlorophyll Index Algorithm: Examples From Taihu Lake and Chaohu Lake. Geoscience and Remote Sensing Letters, IEEE. 2015;PP(99):1136–40. https://doi.org/10.1109/lgrs.2014.2385800
101. Dekker A, Park YJ, Brando V, Schroeder T. Remote sensing of algal blooms with emphasis on Hincksia sordida for the Southeast Queensland coast: a feasibility study. Canberra (Aus): CSIRO Land and Water, 2011.

102. Ahern KS, Ahern CR, Savige GM, Udy JW. Mapping the distribution, biomass and tissue nutrient levels of a marine benthic cyanobacteria bloom (Lyngbya majuscula). Marine And Freshwater Research. 2007; 58(10):883–904. https://doi.org/10.1071/MF07065

103. Robson BJ, Baird M, Wild-Allen K, editors. A physiological model for the marine cyanobacteria, Trichodesmium. MODSIM 2013 -20th International Congress on Modelling and Simulation; 2013 1–6 December; Adelaide, Australia.

104. Baird ME, Cherukuru N, Jones E, Margvelashvili N, Mongin M, Oubelkheir K, et al. Remote-sensing reflectance and true colour produced by a coupled hydrodynamic, optical, sediment, biogeochemical model of the Great Barrier Reef, Australia: Comparison with satellite data. Environmental Modelling & Software. 2016; 78:79–96. https://doi.org/10.1016/j.envsoft.2015.11.025
